# Single‐Cell Transcriptome Profiling Reveals Multicellular Ecosystem of Nucleus Pulposus during Degeneration Progression

**DOI:** 10.1002/advs.202103631

**Published:** 2021-11-26

**Authors:** Ji Tu, Wentian Li, Sidong Yang, Pengyi Yang, Qi Yan, Shenyu Wang, Kaitao Lai, Xupeng Bai, Cenhao Wu, Wenyuan Ding, Justin Cooper‐White, Ashish Diwan, Cao Yang, Huilin Yang, Jun Zou

**Affiliations:** ^1^ Department of Orthopaedic Surgery The First Affiliated Hospital of Soochow University Suzhou 215006 China; ^2^ Spine Labs, St. George and Sutherland Clinical School Faculty of Medicine University of New South Wales Sydney New South Wales 2217 Australia; ^3^ Australian Institute for Bioengineering and Nanotechnology The University of Queensland St. Lucia Brisbane Queensland 4072 Australia; ^4^ Department of Spine Surgery The Third Hospital of Hebei Medical University Shijiazhuang 05000 China; ^5^ Charles Perkins Centre The University of Sydney Sydney NSW 2006 Australia; ^6^ School of Life and Environmental Sciences The University of Sydney Sydney NSW 2006 Australia; ^7^ Computational Systems Biology Group Children's Medical Research Institute Faculty of Medicine and Health The University of Sydney Westmead NSW 2145 Australia; ^8^ The ANZAC Research Institute Concord Repatriation General Hospital Sydney NSW 2139 Australia; ^9^ Concord Clinical School Faculty of Medicine and Health The University of Sydney Sydney NSW 2139 Australia; ^10^ Cancer Care Centre St. George and Sutherland Clinical School Faculty of Medicine University of New South Wales Sydney New South Wales 2052 Australia; ^11^ School of Chemical Engineering The University of Queensland Brisbane Queensland 4072 Australia; ^12^ Spine Service Department of Orthopaedic Surgery St. George Hospital Kogarah New South Wales 2217 Australia; ^13^ Department of Orthopaedic Surgery Wuhan Union Hospital Tongji Medical School Huazhong University of Science and Technology Wuhan Hubei 430022 China

**Keywords:** intervertebral disc degeneration, low back pain, nucleus pulposus, single‐cell RNA sequencing

## Abstract

Although degeneration of the nucleus pulposus (NP) is a major contributor to intervertebral disc degeneration (IVDD) and low back pain, the underlying molecular complexity and cellular heterogeneity remain poorly understood. Here, a comprehensive single‐cell resolution transcript landscape of human NP is reported. Six novel human NP cells (NPCs) populations are identified by their distinct molecular signatures. The potential functional differences among NPC subpopulations are analyzed. Predictive transcripts, transcriptional factors, and signal pathways with respect to degeneration grades are explored. It is reported that fibroNPCs is the subpopulation for end‐stage degeneration. CD90+NPCs are observed to be progenitor cells in degenerative NP tissues. NP‐infiltrating immune cells comprise a previously unrecognized diversity of cell types, including granulocytic myeloid‐derived suppressor cells (G‐MDSCs). Integrin *α*M (CD11b) and oxidized low density lipoprotein receptor 1 (OLR1) as surface markers of NP‐derived G‐MDSCs are uncovered. The G‐MDSCs are found to be enriched in mildly degenerated (grade II and III) NP tissues compared to severely degenerated (grade IV and V) NP tissues. Their immunosuppressive function and alleviation effects on NPCs’ matrix degradation are revealed in vitro. Collectively, this study reveals the NPC‐type complexity and phenotypic characteristics in NP, thereby providing new insights and clues for IVDD treatment.

## Graphical abstract

Low back pain is a common and debilitating condition with degeneration of the nucleus pulposus (NP) as the main cause. Here, human NP tissues with progressive degeneration grades are profiles, and a precise atlas of NP cells (NPCs) and immunocytes is constructed. The dynamics of NPC heterogeneity at the mRNA level during IVDD is characterized. These findings may aid the understanding of molecular complexity and cellular heterogeneity in intervertebral disc degeneration.

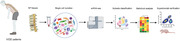

## 1 Introduction

Low back pain is a major disabling health condition in humans, with a lifetime prevalence of as high as 84%.^[^ ^1^ ^]^ The socioeconomic burden of this rheumatologic disorder of the spine is enormous. It was estimated at $85 billion in 2008, and the economic cost has still increased in the last decade.^[^ ^2^ ^]^ Intervertebral disc degeneration (IVDD) is a widely recognized contributor to low back pain.^[^ ^3^ ^]^ The degeneration of the nucleus pulposus (NP), the central gel‐like part of the intervertebral disc, is a significant mechanism of IVDD.^[^ ^4^ ^]^ The current treatments are limited to relieving back or leg symptoms. They do not focus on replenishing the NP loss and restoring the native disk structure. The failure leads to unsatisfactory outcomes, such as recrudescence or degeneration of adjacent motion segments.^[^ ^5^ ^]^ New therapeutic targets are therefore needed.

NP cells (NPCs) are the main cell type residing in the NP, and they are responsible for maintaining tissue homeostasis.^[^ ^6^ ^]^ NPCs proliferate slowly and lack self‐regeneration capacity adding to the intractability of the disease. Current studies on the pathophysiology of NPCs are usually supported by transcriptomic and epigenomic analyses. However, bulk‐tissue level resolution masks the complexity of alterations across cells and within cell types. The uncharacterized cell types and markers residing in the NP raise interest in terms of unexplored cellular heterogeneity.

IVD is the largest avascular organ of the body. Studies addressing immune reactions against the NP have been limited and focused on their detrimental aspects. However, given the complexity of immunity in other immune‐privileged sites,^[^ ^7^ ^]^ immune cell subpopulations may also help restore IVD structure and lessen degeneration. Moreover, intrinsic properties of NPCs, including expression of immunomodulatory factors, and extrinsic microenvironmental changes to immune compartments, remain largely unknown. These issues highlight the importance of understanding the immune panorama of the NP during IVDD pathogenesis.

Single‐cell RNA sequencing (scRNA‐seq) provides a powerful alternative to study the cellular heterogeneity of NP tissues. Here, we aimed to provide a single‐cell view of IVDD pathology, profiling 39 732 cells from NP tissues across eight individuals with different grades of progressive degeneration. Notably, we comprehensively characterized the transcriptome feature of NPCs and immune cells, and we decoded the cell percentage, the heterogeneity of cell subtypes during degeneration, providing a unique cellular‐level insight into transcriptional alterations associated with IVDD pathology.

## 2 Results

### 2.1 Single‐Cell Profiling of NPC Atlas in Human Subjects with IVDD Pathology

**Figure 1:**
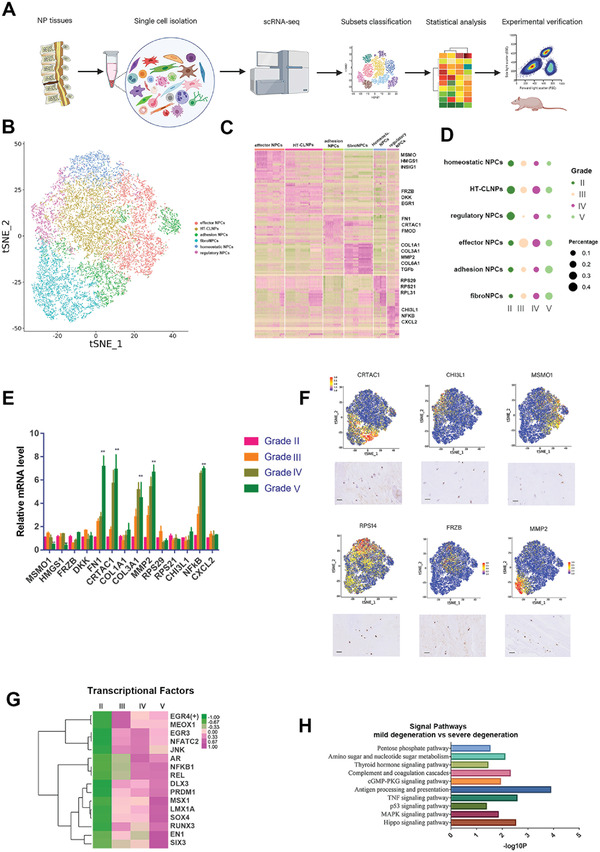
Identification of human NPC atlas and transcriptional changes correlated with IVDD severity. A) Graphical representation of the experimental workflow. B) UMAP visualization of human NP cells identified six different clusters after unsupervised clustering. Each dot corresponds to one single cell colored according to cell cluster. C) Heatmap revealing the scaled expression of differentially expressed genes for each cluster. D) Dot plots showing the grade distribution in each NP cell subsets. E) RT‐qPCR for the representative genes of NPC atlas in different degenerative grades discs (*n* = 3 with mean ± SD shown). F) Representative immunohistochemistry assay of indicated genes in NP tissues. G) Heatmap showing grade‐related transcription factors. H) Enriched signal pathways related with degeneration grades.

We dissociated NP tissues from eight IVDD patients (**Table** **1**) with different degrees of degeneration (see the Experimental Section) and performed scRNA‐seq on the BD Rhapsody system (**Figure** **1**A and Figure S1A, Supporting Information). After quality control and doublet exclusion filtering to remove cells with low gene detection (<600 genes) and high mitochondrial gene content (>8%) (Figure S1E–H, Supporting Information), NPCs were identified based on their levels of the transcripts that encode different proteins [e.g., aggrecan proteoglycan (ACAN) and SRY‐box transcription factor 9 (SOX9)]. Consistent with previous studies,^[^ ^8^ ^]^ we found a lack of distinctive clusters in the tSNE map (Figure 1B) which may suggest the heterogeneity exhibited from these cell populations. Six subpopulations were identified based on their highly expressed genes and published single‐cell studies (Figure 1C):

1) hypertrophy chondrocyte‐like NPCs (HT‐CLNPs; Groups 0, 1; expressing FRZB, DKK);^[^ ^9^ ^]^
2) effector NPCs (Groups 2 and 4, expressing mRNAs that encode proteins that participate in genes cellular metabolism, e.g., MSMO1^[^ ^10^ ^]^ and HMGCS1^[^ ^11^ ^]^);
3) homeostatic NPCs (Group 5; expressing RPS29 and RPS21);^[^ ^8b^ ^]^
4) regulatory NPCs (Group 6; expressing CHI3L1,^[^ ^8b^ ^]^ CXCL2, and NFKB^[^ ^12^ ^]^);
5) fibroNPCs (Groups 8, 9, and 10, expressing mRNAs that encode related with fibrosis, COL1A1, COL3A1, and COL6A1); and^[^ ^8^ ^]^
6) adhesion NPCs (Groups 3 and 11; expressing mRNAs that related to cell adhesion and migration, such as FN1^[^ ^13^ ^]^ and CRTAC1^[^ ^14^ ^]^).

**Table 1:** Basic information and characteristics for participants

Patient ID	Age [years]	Gender	Weight [kg]	Reason for surgery	Pfirrmann grading	CRP [mg dL^−1^]	WBC (109 L^−1^)	Lymphocyte (109 L^−1^)	Monocyte (109 L^−1^)
S1	63	Male	60	Burst fracture	II	0.047	6.46	2.16	0.64
S2	41	Male	73.5	Burst fracture	II	0.706	11.37	2.35	1.02
S3	56	Female	62	Lumbar disc herniation	III	0.079	7.25	1.63	0.47
S4	65	Female	76	Lumbar disc herniation	III	0.154	6.78	2.15	0.54
S5	64	Female	50	Lumbar disc herniation	IV	0.027	4.83	1.55	0.19
S6	53	Female	60	Lumbar disc herniation	IV	0.184	5.53	2.28	0.49
S7	54	Male	68	Lumbar disc herniation	V	0.011	10.79	3.55	0.83
S8	56	Male	55	Lumbar disc herniation	V	0.022	5.75	1.86	0.61

Patient ID	Blood glucose [mmol L^−1^]	ALT [U L^−1^]	AST [U L^−1^]	Total protein [g L^−1^]	Serum albumin [g L^−1^]	BUN [mmol L^−1^]	Cre [μmoI L^−1^]	TT [s]	APTT [s]	INR
S1	3.77	7.4	12.7	57.3	37.4	3.3	42.4	21.6	26.7	1.01
S2	5.40	27.7	13.4	63.3	41.0	5.0	59.1	18.4	22.8	0.92
S3	5.05	18.7	14.8	73.3	44.9	4.8	46.4	18.1	21.9	1.02
S4	5.41	10.6	12.3	66.5	42.1	6.1	38.0	19.8	24.4	1.07
S5	4.52	13.6	12.6	69.0	44.0	5.5	49.1	19.5	26.2	1.00
S6	4.66	26.5	18.3	66.3	40.7	4.6	53.8	19.7	22.4	0.99
S7	4.84	20.6	18.0	71.5	46.3	4.9	60.3	18.2	23.6	0.96
S8	4.70	33.7	17.0	65.8	39.4	6.4	82.0	18.6	25.9	0.92

We next analyzed the relationship between the degeneration grades and distributions of the cell populations. HT‐CLNPs and regulatory NPCs were the main cells for grade II discs. Effector NPCs and HT‐CLNPs were major subpopulations in grades III and IV discs. For grade V discs, fibroNPCs and adhesion NPs were the main NPCs (Figure 1D). Moreover, the proportion of adhesion NPCs and fibroNPCs increased with the severity of IVDD (from grade II to V), while the proportion of homeostatic NPCs showed opposite trends.

Real‐time quantitative PCR (qPCR) was used to validate the indicated gene expression in NPCs from different degeneration grades. The results revealed that the expressions of FN1 and CRTAC1, COL3A1, and MMP2 (markers of adhesion NPCs and fibroNPCs) were significantly elevated in NPCs from late‐stage degenerative discs (grade IV and V). The expressions of homeostatic NPC markers, MSMO1 and HMGS1, significantly decreased in NPCs from late‐stage degenerative discs. (Figure 1E). We then performed an immunohistochemistry assay to validate the expressions of markers for each NPC subpopulation at the protein level (Figure 1F).

### 2.2 Identification of NPC Transcriptional Changes Correlated with IVDD Severity

A group of transcriptional factors (TFs) were identified to be related to grade of degeneration (Figure 1G). Some TFs, like AR, REL, LMX1A, and PRDM1, were first revealed. Enrichment for TFs of REL in grade IV and V discs may be related to Rel/NF‐*κ*B signal transduction pathway in IVDD.^[^ ^15^ ^]^

Besides, we compared differentially expressed genes (DEGs) between severe (grades IV and V) and mild (grades II and III) degeneration and identified candidate genes related to IVDD progression, including HSPH1, CTGF, MMP13, and HSP90AA1 (**Table** **2**). There are significantly elevated expressions of a list of heat shock protein (HSP) genes in severe degenerative discs, including HSPA1B, HSPH1, HSP90AA1, HSPA8, HSPA1A, HSPB8, and HSPD1, indicating the importance of stress‐related mechanisms in IVDD. Enrichment for TF, SOX4, and INHBA genes is consistent with the importance of TGF‐*β* signaling in IVDD.^[^ ^16^ ^]^ ANXA5 was also elevated, which may be related to mitochondrial dysfunction induced cell apoptosis.^[^ ^17^ ^]^

**Table 2:** DEGs by comparing severe degeneration grade (Grade III and IV) to mild degeneration grade (Grade I and II)

Upregulated genes					
Gene	*P* value	avg_logFC	pct.1	pct.2	p_val_adj
INHBA	0	1.542242	0.787	0.491	0
FHL2	0	1.355454	0.65	0.274	0
KLHL21	0	1.150942	0.633	0.334	0
CYR61	0	1.149118	0.947	0.887	0
HSPA1B	0	1.059508	0.868	0.733	0
NFATC2	0	1.027662	0.497	0.173	0
HSPH1	0	0.983483	0.813	0.634	0
CTGF	0	0.979719	0.957	0.877	0
MMP13	0	0.948365	0.822	0.626	0
HSP90AA1	0	0.899521	0.964	0.896	0
RTN4	0	0.759251	0.836	0.694	0
HSPA8	0	0.650494	0.882	0.8	0
ACTG1	0	0.627876	0.979	0.96	0
ANXA5	0	0.608453	0.908	0.839	0
LUM	0	0.58595	0.984	0.97	0
H3F3B	0	0.496407	0.963	0.94	0
HSPA1A	5.2 × 10^−284^	0.735349	0.91	0.866	1.1 × 10^−279^
GFPT2	6.9 × 10^−261^	0.734121	0.682	0.489	1.5 × 10^−256^
KLF4	7 × 10^−258^	0.702163	0.905	0.846	1.5 × 10^−253^
ATP1B1	6.7 × 10^−252^	0.710599	0.614	0.392	1.5 × 10^−247^
PCOLCE2	1.2 × 10^−250^	0.563587	0.869	0.752	2.6 × 10^−246^
EMP1	7.9 × 10^−244^	0.770794	0.907	0.84	1.7 × 10^−239^
SLC39A14	9.2 × 10^−242^	0.606197	0.801	0.68	2 × 10^−236^
HERPUD1	1.3 × 10^−237^	0.515521	0.873	0.801	2.8 × 10^−233^
PLOD2	5.6 × 10^−227^	0.471544	0.866	0.805	1.2 × 10^−222^
HSPB8	1.8 × 10^−224^	0.780165	0.687	0.502	4 × 10^−220^
ZSWIM6	1.8 × 10^−219^	0.548961	0.309	0.088	3.8 × 10^−215^
BTF3	3.4 × 10^−219^	0.457349	0.863	0.785	7.4 × 10^−215^
ANXA1	2.2 × 10^−216^	0.58282	0.928	0.894	4.7 × 10^−212^
ACKR3	4.6 × 10^−215^	0.909398	0.595	0.397	1 × 10^−210^
MYADM	1.2 × 10^−214^	0.682027	0.739	0.614	2.5 × 10^−210^
ACTB	1.6 × 10^−214^	0.357681	0.965	0.945	3.4 × 10^−210^
AMOTL2	1.1 × 10^−212^	0.883341	0.388	0.169	2.5 × 10^−208^
FOXC2	1.9 × 10^−210^	0.758533	0.43	0.208	4.2 × 10^−206^
TXN	1.2 × 10^−201^	0.630013	0.708	0.571	2.6 × 10^−197^
UAP1	7.7 × 10^−201^	0.602079	0.708	0.556	1.7 × 10^−196^
TM4SF1	4.7 × 10^−200^	0.936974	0.488	0.282	1 × 10^−195^
RYBP	1.2 × 10^−199^	0.648708	0.654	0.479	2.7 × 10^−195^
FMOD	1.1 × 10^−195^	0.535043	0.908	0.89	2.3 × 10^−191^
RPL30	1.8 × 10^−186^	0.341223	0.949	0.941	3.9 × 10^−182^
GPRC5A	7.1 × 10^−186^	0.670452	0.596	0.403	1.5 × 10^−181^
HSPD1	1.2 × 10^−184^	0.574874	0.775	0.666	2.7 × 10^−180^
FN1	4.3 × 10^−180^	0.740568	0.968	0.95	9.4 × 10^−176^
TMED2	2.4 × 10^−177^	0.583225	0.672	0.564	5.2 × 10^−173^
RAN	1.5 × 10^−174^	0.462583	0.799	0.715	3.3 × 10^−170^
SERPINE2	2 × 10^−174^	0.721224	0.845	0.773	4.4 × 10^−170^
CTNNB1	1.9 × 10^−167^	0.511848	0.66	0.526	4.2 × 10^−163^
IL11	7.6 × 10^−165^	0.928692	0.287	0.1	1.6 × 10^−160^
OAT	1 × 10^−163^	0.473536	0.728	0.608	2.3 × 10^−159^
CD55	6.3 × 10^−161^	0.717146	0.707	0.576	1.4 × 10^−156^

Downregulated genes					
Gene	*P*‐value	avg_logFC	pct.1	pct.2	p_val_adj
CCL4	0	−0.28029	0.019	0.2	0
CCL3	0	−0.28546	0.022	0.194	0
BARX1	0	−0.28722	0.004	0.148	0
CCL3L3	0	−0.30709	0.026	0.22	0
SLC25A1	0	−0.32756	0.009	0.169	0
C19orf70	0	−0.35763	0.008	0.172	0
EIF3G	0	−0.36931	0.014	0.211	0
AIP	0	−0.38403	0.009	0.196	0
GNB2	0	−0.38455	0.029	0.241	0
NT5DC2	0	−0.38716	0.03	0.228	0
ASS1	0	−0.39016	0.047	0.25	0
RFNG	0	−0.39021	0.052	0.263	0
LAMTOR4	0	−0.40186	0.059	0.274	0
CCL4L2	0	−0.40452	0.017	0.249	0
SORBS3	0	−0.40863	0.042	0.261	0
ARL6IP4	0	−0.41084	0.031	0.241	0
HLA‐DRA	0	−0.42087	0.016	0.26	0
NRBP1	0	−0.42194	0.048	0.277	0
HLA‐DRB1	0	−0.44289	0.012	0.249	0
YIF1A	0	−0.45561	0.053	0.286	0
SH3BGRL3	0	−0.46366	0.044	0.258	0
ATP6V0B	0	−0.46735	0.091	0.334	0
GUK1	0	−0.48434	0.049	0.281	0
ANAPC11	0	−0.49102	0.087	0.323	0
H2AFJ	0	−0.49279	0.034	0.256	0
COX5B	0	−0.49904	0.077	0.305	0
GAS6	0	−0.5001	0.028	0.244	0
MYL9	0	−0.50117	0.029	0.238	0
BSG	0	−0.50177	0.095	0.37	0
METTL26	0	−0.50619	0.028	0.274	0
LY6E	0	−0.50741	0.039	0.281	0
ADIRF	0	−0.5311	0.03	0.264	0
MMP24OS	0	−0.54508	0.042	0.304	0
TUBB4B	0	−0.5488	0.112	0.379	0
LAMB2	0	−0.55969	0.095	0.35	0
NOP53	0	−0.56125	0.119	0.408	0
PHPT1	0	−0.56333	0.013	0.281	0
RCN3	0	−0.56512	0.164	0.433	0
RPS5	0	−0.56633	0.817	0.898	0
GPX1	0	−0.57485	0.108	0.38	0
NUCB1	0	−0.58197	0.217	0.506	0
FXYD1	0	−0.59014	0.033	0.28	0
PRDX2	0	−0.59675	0.076	0.353	0
RPL3	0	−0.60441	0.242	0.558	0
EIF5A	0	−0.60456	0.076	0.392	0
FXYD5	0	−0.61747	0.175	0.461	0
ID3	0	−0.62269	0.177	0.488	0
LTBP3	0	−0.62545	0.088	0.376	0
MDFI	0	−0.63195	0.035	0.267	0
ALDOA	0	−0.63467	0.489	0.722	0
RPL13	0	−0.63534	0.775	0.892	0

Based on a IVDD grade‐related gene set, a group of signaling pathways were found to potentially promote degeneration, including antigen processing and presentation, the TNF pathway, MAPK, and Hippo pathways (Figure 1H).

### 2.3 ScRNA‐seq Reveals Transcriptional Features of NPCs Subpopulations

**Figure 2:**
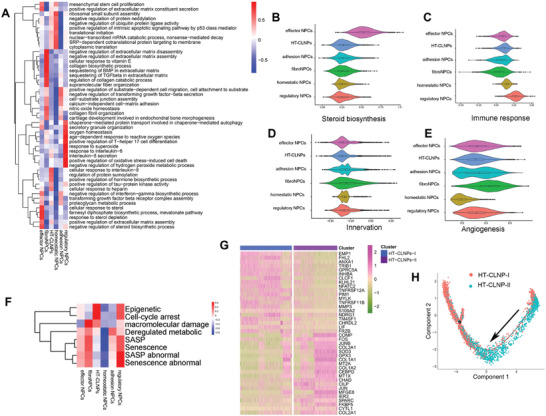
ScRNA‐seq reveals transcriptional features of NPCs subpopulations. A) QuSAGE analysis of cell subpopulation specific differential expression colored by statistically significant normalized enrichment scores. B–E) Violin plots of steroid biosynthesis, immune response, innervation, and angiogenesis score for each cluster. F) Correlation of scRNA‐seq defined NPCs subpopulations with cell senescence. G) Heatmap showing the scaled expression of the differentially expressed genes (DEGs) for HT‐CLNP‐I and HT‐CLNP‐II subsets. H) Pseudotime trajectory axis revealing the progression of HT‐CLNP‐I and HT‐CLNP‐II.

To analyze the functional differences among subpopulations, we used the Quantitative Set Analysis for Gene Expression (QuSAGE) (**Figure** **2**A–C) and Gene Ontology (GO) database (**Table** **3**)^[^ ^18^ ^]^ to investigate the biological process of our identified NPCs subpopulations. Effector NPCs were enriched with metabolic process‐related genes (e.g., sterol biosynthesis and glycosaminoglycan metabolism) and positive regulation of extracellular matrix (ECM) assembly. Regulatory NPCs were enriched with highly expressed genes responsible for cellular responses to inflammation and endogenous stimuli, such as CXCL3, IL‐6, and CHI3L1, indicating that these cells might potentially regulate immune functions. Homeostatic NPCs were enriched for processes related to cellular homeostasis, including translational regulation and protein/RNA metabolism. Adhesion NPCs were enriched for cell migration and cell–matrix adhesion.

**Table 3:** GO Terms for NP subpopulations

Efector NPCs GO terms		
GOID	GOTerm	*P‐*value
GO:0016126	sterol biosynthetic process	2.432 × 10^−11^
GO:0006695	cholesterol biosynthetic process	5.795 × 10^−10^
GO:0030198	extracellular matrix organization	3.136 × 10^−8^
GO:0044281	small molecule metabolic process	4.528 × 10^−8^
GO:0001558	regulation of cell growth	2.285 × 10^−6^
GO:0001501	skeletal system development	3.133 × 10^−6^
GO:0006694	steroid biosynthetic process	5.308 × 10^−6^
GO:0008203	cholesterol metabolic process	9.283 × 10^−6^
GO:0008202	steroid metabolic process	1.061 × 10^−5^
GO:0006048	UDP‐N‐acetylglucosamine biosynthetic process	1.904 × 10^−5^
GO:0008299	isoprenoid biosynthetic process	2.17 × 10^−5^
GO:0010628	positive regulation of gene expression	3.007 × 10^−5^
GO:0008285	negative regulation of cell proliferation	3.96 × 10^−5^
GO:0009405	pathogenesis	5.295 × 10^−5^
GO:0072593	reactive oxygen species metabolic process	6.08 × 10^−5^
GO:0006629	lipid metabolic process	9.184 × 10^−5^
GO:0048661	positive regulation of smooth muscle cell proliferation	9.297 × 10^−5^
GO:0033173	calcineurin‐NFAT signaling cascade	0.0001037
GO:0030199	collagen fibril organization	0.0001053
GO:0042127	regulation of cell proliferation	0.0001366
GO:0001525	angiogenesis	0.0001439
GO:0014070	response to organic cyclic compound	0.0001497
GO:0010955	negative regulation of protein processing	0.0001541
GO:0042340	keratan sulfate catabolic process	0.0001541
GO:0006011	UDP‐glucose metabolic process	0.0001563
GO:0007568	aging	0.0001871
GO:0006633	fatty acid biosynthetic process	0.0002058
GO:0043434	response to peptide hormone	0.000223
GO:0055114	oxidation‐reduction process	0.0002321
GO:0005975	carbohydrate metabolic process	0.0003237
GO:0043065	positive regulation of apoptotic process	0.0003622
GO:0030203	glycosaminoglycan metabolic process	0.0003728
GO:0043066	negative regulation of apoptotic process	0.000445
GO:0003331	positive regulation of extracellular matrix constituent secretion	0.0004652
GO:0006065	UDP‐glucuronate biosynthetic process	0.0004652
GO:2000544	regulation of endothelial cell chemotaxis to fibroblast growth factor	0.0004652
GO:0042542	response to hydrogen peroxide	0.0004732
GO:0032270	positive regulation of cellular protein metabolic process	0.0005055
GO:0006915	apoptotic process	0.0006357
GO:0010035	response to inorganic substance	0.0006375
GO:0008284	positive regulation of cell proliferation	0.0006802
GO:0044255	cellular lipid metabolic process	0.0007222
GO:0060591	chondroblast differentiation	0.0009226
GO:1903169	regulation of calcium ion transmembrane transport	0.0009226
GO:0006933	negative regulation of cell adhesion involved in substrate‐bound cell migration	0.0009226
GO:0051387	negative regulation of neurotrophin TRK receptor signaling pathway	0.0009226
GO:0009314	response to radiation	0.0009242
GO:0060291	long‐term synaptic potentiation	0.0010297
GO:0045444	fat cell differentiation	0.0013192
GO:0031394	positive regulation of prostaglandin biosynthetic process	0.001525

FibroNPCs GO terms		
GOID	GOTerm	*P*‐value
GO:0030198	extracellular matrix organization	8.663 × 10^−42^
GO:0007155	cell adhesion	9.642 × 10^−28^
GO:0022617	extracellular matrix disassembly	1.776 × 10^−23^
GO:0030574	collagen catabolic process	5.312 × 10^−19^
GO:0007411	axon guidance	2.226 × 10^−16^
GO:0030199	collagen fibril organization	8.14 × 10^−16^
GO:0001525	angiogenesis	1.304 × 10^−12^
GO:0002576	platelet degranulation	1.732 × 10^−11^
GO:0030168	platelet activation	9.024 × 10^−11^
GO:0007507	heart development	1.504 × 10^−10^
GO:0006928	cellular component movement	2.848 × 10^−10^
GO:0007596	blood coagulation	2.876 × 10^−9^
GO:0008285	negative regulation of cell proliferation	7.397 × 10^−9^
GO:0016477	cell migration	3.908 × 10^−7^
GO:0001558	regulation of cell growth	4.529 × 10^−7^
GO:0009612	response to mechanical stimulus	6.42 × 10^−7^
GO:0048010	vascular endothelial growth factor receptor signaling pathway	1.441 × 10^−6^
GO:0051017	actin filament bundle assembly	1.628 × 10^−6^
GO:0001649	osteoblast differentiation	2.019 × 10^−6^
GO:0001666	response to hypoxia	2.039 × 10^−6^
GO:0008283	cell proliferation	2.174 × 10^−6^
GO:0035987	endodermal cell differentiation	3.631 × 10^−6^
GO:0007162	negative regulation of cell adhesion	3.942 × 10^−6^
GO:0007160	cell‐matrix adhesion	4.348 × 10^−6^
GO:0030336	negative regulation of cell migration	4.63 × 10^−6^
GO:0051764	actin crosslink formation	5.041 × 10^−6^
GO:0071230	cellular response to amino acid stimulus	5.525 × 10^−6^
GO:0050900	leukocyte migration	6.418 × 10^−6^
GO:0048013	ephrin receptor signaling pathway	7.117 × 10^−6^
GO:0030335	positive regulation of cell migration	7.697 × 10^−6^
GO:0001974	blood vessel remodeling	8.644 × 10^−6^
GO:0016525	negative regulation of angiogenesis	9 × 10^−6^
GO:0001501	skeletal system development	9.137 × 10^−6^
GO:0071305	cellular response to vitamin D	1.097 × 10^−5^
GO:0010811	positive regulation of cell‐substrate adhesion	1.102 × 10^−5^
GO:0045669	positive regulation of osteoblast differentiation	1.291 × 10^−5^
GO:0048813	dendrite morphogenesis	1.394 × 10^−5^
GO:0051493	regulation of cytoskeleton organization	1.57 × 10^−5^
GO:0042060	wound healing	1.585 × 10^−5^
GO:0009611	response to wounding	1.821 × 10^−5^
GO:0006936	muscle contraction	2.038 × 10^−5^
GO:0006987	activation of signaling protein activity involved in unfolded protein response	2.151 × 10^−5^
GO:0043491	protein kinase B signaling	2.758 × 10^−5^
GO:0046718	viral entry into host cell	3.305 × 10^−5^
GO:0001938	positive regulation of endothelial cell proliferation	3.468 × 10^−5^
GO:0032964	collagen biosynthetic process	3.531 × 10^−5^
GO:0060674	placenta blood vessel development	3.762 × 10^−5^
GO:0031532	actin cytoskeleton reorganization	5.90377 × 10^−5^
GO:0006898	receptor‐mediated endocytosis	6.00706 × 10^−5^
GO:0030036	actin cytoskeleton organization	6.00706 × 10^−5^

Homestatic NPs GO terms		
GOID	GOTerm	*P*‐value
GO:0006614	SRP‐dependent cotranslational protein targeting to membrane	2.963 × 10^−89^
GO:0006415	translational termination	4.999 × 10^−86^
GO:0006414	translational elongation	7.153 × 10^−80^
GO:0019083	viral transcription	1.067 × 10^−76^
GO:0006412	translation	1.077 × 10^76^
GO:0000184	nuclear‐transcribed mRNA catabolic process, nonsense‐mediated decay	5.322 × 10^−75^
GO:0019058	viral life cycle	9.812 × 10^−75^
GO:0006413	translational initiation	1.227 × 10^−74^
GO:0016070	RNA metabolic process	1.437 × 10^−71^
GO:0016071	mRNA metabolic process	3.898 × 10^−67^
GO:0010467	gene expression	9.55 × 10^−63^
GO:0016032	viral process	4.056 × 10^−53^
GO:0044267	cellular protein metabolic process	3.626 × 10^−47^
GO:0022904	respiratory electron transport chain	2.26 × 10^−21^
GO:0044237	cellular metabolic process	1.722 × 10^−18^
GO:0002181	cytoplasmic translation	5.217 × 10^−15^
GO:0042274	ribosomal small subunit biogenesis	1.254 × 10^−11^
GO:0000027	ribosomal large subunit assembly	1.824 × 10^−11^
GO:0000028	ribosomal small subunit assembly	2.147 × 10^−10^
GO:0002479	antigen processing and presentation of exogenous peptide antigen via MHC class I, TAP‐dependent	1.431 × 10^−9^
GO:0042590	antigen processing and presentation of exogenous peptide antigen via MHC class I	2.655 × 10^−9^
GO:0006120	mitochondrial electron transport, NADH to ubiquinone	1.106 × 10^−8^
GO:0006364	rRNA processing	1.348 × 10^−8^
GO:1902600	hydrogen ion transmembrane transport	1.963 × 10^−8^
GO:0006367	transcription initiation from RNA polymerase II promoter	2.211 × 10^−8^
GO:0051437	positive regulation of ubiquitin‐protein ligase activity involved in mitotic cell cycle	2.365 × 10^−8^
GO:0051439	regulation of ubiquitin‐protein ligase activity involved in mitotic cell cycle	4.824 × 10^−8^
GO:0000398	mRNA splicing, via spliceosome	5.565 × 10^−8^
GO:0051436	negative regulation of ubiquitin‐protein ligase activity involved in mitotic cell cycle	9.745 × 10^−8^
GO:0038061	NIK/NF‐kappaB signaling	1.169 × 10^−7^
GO:0031145	anaphase‐promoting complex‐dependent proteasomal ubiquitin‐dependent protein catabolic process	1.282 × 10^−7^
GO:0002474	antigen processing and presentation of peptide antigen via MHC class I	2.331 × 10^−7^
GO:0006977	DNA damage response, signal transduction by p53 class mediator resulting in cell cycle arrest	8.067 × 10^−7^
GO:0033209	tumor necrosis factor‐mediated signaling pathway	9.747 × 10^−7^
GO:0000387	spliceosomal snRNP assembly	2.742 × 10^−6^
GO:0034660	ncRNA metabolic process	4.53 × 10^−6^
GO:0006521	regulation of cellular amino acid metabolic process	5.739 × 10^−6^
GO:0050434	positive regulation of viral transcription	6.873 × 10^−6^
GO:0042776	mitochondrial ATP synthesis coupled proton transport	1.66 × 10^−5^
GO:0044281	small molecule metabolic process	1.913 × 10^−5^
GO:0006446	regulation of translational initiation	2.391 × 10^−5^
GO:0000082	G1/S transition of mitotic cell cycle	2.8 × 10^−5^
GO:0010499	proteasomal ubiquitin‐independent protein catabolic process	3.292 × 10^−5^
GO:0019068	virion assembly	3.292 × 10^−5^
GO:0000209	protein polyubiquitination	3.844 × 10^−5^
GO:0042254	ribosome biogenesis	4.293 × 10^−5^
GO:0001731	formation of translation preinitiation complex	4.472 × 10^−5^
GO:0006368	transcription elongation from RNA polymerase II promoter	4.798 × 10^−5^
GO:0021888	hypothalamus gonadotrophin‐releasing hormone neuron development	4.944 × 10^−5^
GO:0002223	stimulatory C‐type lectin receptor signaling pathway	5.147 × 10^−5^

HT‐CLNPs GO terms		
GOID	GOTerm	*P*‐value
GO:0043066	negative regulation of apoptotic process	9.969 × 10^−11^
GO:0044267	cellular protein metabolic process	1.093 × 10^−9^
GO:0000122	negative regulation of transcription from RNA polymerase II promoter	1.504 × 10^−9^
GO:0006413	translational initiation	2.324 × 10^−9^
GO:0045944	positive regulation of transcription from RNA polymerase II promoter	2.996E‐09
GO:0006366	transcription from RNA polymerase II promoter	4.917 × 10^−9^
GO:0010467	gene expression	1.639 × 10^−7^
GO:0007623	circadian rhythm	3.873 × 10^−7^
GO:0001501	skeletal system development	4.605 × 10^−7^
GO:1901653	cellular response to peptide	8.175 × 10^−7^
GO:0043065	positive regulation of apoptotic process	1.082 × 10^−6^
GO:0007179	transforming growth factor beta receptor signaling pathway	4.329 × 10^−6^
GO:0009409	response to cold	6.217 × 10^−6^
GO:0006986	response to unfolded protein	6.218 × 10^−6^
GO:0006446	regulation of translational initiation	7.305 × 10^−6^
GO:0006412	translation	8.13 × 10^−6^
GO:0016071	mRNA metabolic process	8.787 × 10^−6^
GO:0009612	response to mechanical stimulus	1.109 × 10^−5^
GO:0032496	response to lipopolysaccharide	1.285 × 10^−5^
GO:0008284	positive regulation of cell proliferation	2.057 × 10^−5^
GO:0035914	skeletal muscle cell differentiation	2.28 × 10^−5^
GO:0071479	cellular response to ionizing radiation	2.342 × 10^−5^
GO:0014070	response to organic cyclic compound	2.375 × 10^−5^
GO:0030968	endoplasmic reticulum unfolded protein response	2.616 × 10^−5^
GO:0070301	cellular response to hydrogen peroxide	4.16 × 10^−5^
GO:0006415	translational termination	5.61 × 10^−5^
GO:0006414	translational elongation	6.171 × 10^−5^
GO:0006355	regulation of transcription, DNA‐templated	7.051 × 10^−5^
GO:0006351	transcription, DNA‐templated	7.28 × 10^−5^
GO:0006457	protein folding	7.913 × 10^−5^
GO:0051726	regulation of cell cycle	8.277 × 10^−5^
GO:0042493	response to drug	8.474 × 10^−5^
GO:0010501	RNA secondary structure unwinding	9.475 × 10^−5^
GO:0006915	apoptotic process	9.491 × 10^−5^
GO:0006950	response to stress	9.576 × 10^−5^
GO:0034097	response to cytokine	0.0001053
GO:0006987	activation of signaling protein activity involved in unfolded protein response	0.0001053
GO:0071499	cellular response to laminar fluid shear stress	0.0001053
GO:0000398	mRNA splicing, via spliceosome	0.0001095
GO:0048705	skeletal system morphogenesis	0.0001226
GO:0071353	cellular response to interleukin‐4	0.0001237
GO:0016070	RNA metabolic process	0.0001291
GO:0043433	negative regulation of sequence‐specific DNA binding transcription factor activity	0.0001513
GO:0002486	antigen processing and presentation of endogenous peptide antigen via MHC class I via ER pathway, TAP‐independent	0.000158
GO:0045892	negative regulation of transcription, DNA‐templated	0.0001617
GO:0048662	negative regulation of smooth muscle cell proliferation	0.0002132
GO:0008380	RNA splicing	0.0002219
GO:0043434	response to peptide hormone	0.0002296
GO:0021762	substantia nigra development	0.0002719
GO:0034976	response to endoplasmic reticulum stress	0.00029

Adhesion NPs GO terms		
GOID	GOTerm	*P*‐value
GO:0030198	extracellular matrix organization	1.294 × 10^−17^
GO:0007155	cell adhesion	2.035 × 10^−10^
GO:0030199	collagen fibril organization	2.478 × 10^−9^
GO:0001501	skeletal system development	8.734 × 10^−9^
GO:0045944	positive regulation of transcription from RNA polymerase II promoter	1.663 × 10^−8^
GO:0022617	extracellular matrix disassembly	3.188 × 10^−7^
GO:0000122	negative regulation of transcription from RNA polymerase II promoter	6.322 × 10^−7^
GO:0030574	collagen catabolic process	1.183 × 10^−6^
GO:0007050	cell cycle arrest	1.66 × 10^−6^
GO:0035987	endodermal cell differentiation	7.714 × 10^−6^
GO:0007179	transforming growth factor beta receptor signaling pathway	8.171 × 10^−6^
GO:0001525	angiogenesis	1.4 × 10^−5^
GO:0001568	blood vessel development	1.842 × 10^−5^
GO:0006865	amino acid transport	2.386 × 10^−5^
GO:0030512	negative regulation of transforming growth factor beta receptor signaling pathway	3.834 × 10^−5^
GO:0045444	fat cell differentiation	4.298 × 10^−5^
GO:0048146	positive regulation of fibroblast proliferation	5.614 × 10^−5^
GO:0006366	transcription from RNA polymerase II promoter	5.619 × 10^−5^
GO:0043001	Golgi to plasma membrane protein transport	6.09 × 10^−5^
GO:0003007	heart morphogenesis	6.397 × 10^−5^
GO:0014912	negative regulation of smooth muscle cell migration	6.773 × 10^−5^
GO:0003333	amino acid transmembrane transport	7.054 × 10^−5^
GO:1902966	positive regulation of protein localization to early endosome	7.632 × 10^−5^
GO:0001503	ossification	0.000107
GO:0045599	negative regulation of fat cell differentiation	0.0001124
GO:0048705	skeletal system morphogenesis	0.0001124
GO:0032956	regulation of actin cytoskeleton organization	0.00013
GO:0046825	regulation of protein export from nucleus	0.0001504
GO:0061028	establishment of endothelial barrier	0.0001781
GO:0043277	apoptotic cell clearance	0.0001781
GO:0007219	Notch signaling pathway	0.0001879
GO:0010628	positive regulation of gene expression	0.0002892
GO:0050919	negative chemotaxis	0.000301
GO:0048008	platelet‐derived growth factor receptor signaling pathway	0.0003392
GO:0008284	positive regulation of cell proliferation	0.0003679
GO:0048593	camera‐type eye morphogenesis	0.0003809
GO:0060325	face morphogenesis	0.0003956
GO:0035989	tendon development	0.0003967
GO:0071709	membrane assembly	0.0003967
GO:1903225	negative regulation of endodermal cell differentiation	0.0003967
GO:0030335	positive regulation of cell migration	0.0003991
GO:0002053	positive regulation of mesenchymal cell proliferation	0.0004587
GO:0030154	cell differentiation	0.0004762
GO:0060021	palate development	0.0004797
GO:0007229	integrin‐mediated signaling pathway	0.0005359
GO:0045893	positive regulation of transcription, DNA‐templated	0.0005761
GO:0042340	keratan sulfate catabolic process	0.0006041
GO:0051216	cartilage development	0.0006198

Regulatory NPCs GO terms		
GOID	GOTerm	*P*‐value
GO:0006954	inflammatory response	4.936 × 10^−13^
GO:0071294	cellular response to zinc ion	3.004 × 10^−9^
GO:0045926	negative regulation of growth	9.96 × 10^−9^
GO:0035666	TRIF‐dependent toll‐like receptor signaling pathway	2 × 10^−8^
GO:0071222	cellular response to lipopolysaccharide	2.027 × 10^−8^
GO:0002756	MyD88‐independent toll‐like receptor signaling pathway	2.248 × 10^−8^
GO:0034138	toll‐like receptor 3 signaling pathway	3.161 × 10^−8^
GO:0034142	toll‐like receptor 4 signaling pathway	1.434 × 10^−7^
GO:0071276	cellular response to cadmium ion	1.933 × 10^−7^
GO:0032496	response to lipopolysaccharide	3.658 × 10^−7^
GO:0002224	toll‐like receptor signaling pathway	4.694 × 10^−7^
GO:0010628	positive regulation of gene expression	5.726 × 10^−7^
GO:0034166	toll‐like receptor 10 signaling pathway	1.709 × 10^−6^
GO:0034146	toll‐like receptor 5 signaling pathway	1.709 × 10^−6^
GO:0048711	positive regulation of astrocyte differentiation	1.771 × 10^−6^
GO:0008285	negative regulation of cell proliferation	1.972 × 10^−6^
GO:0038124	toll‐like receptor TLR6:TLR2 signaling pathway	3.095 × 10^−6^
GO:0038123	toll‐like receptor TLR1:TLR2 signaling pathway	3.095 × 10^−6^
GO:0034162	toll‐like receptor 9 signaling pathway	3.398 × 10^−6^
GO:0034134	toll‐like receptor 2 signaling pathway	3.726 × 10^−6^
GO:0006915	apoptotic process	3.786 × 10^−6^
GO:0070374	positive regulation of ERK1 and ERK2 cascade	4.314 × 10^−6^
GO:0070301	cellular response to hydrogen peroxide	5.255 × 10^−6^
GO:0002755	MyD88‐dependent toll‐like receptor signaling pathway	6.844 × 10^−6^
GO:0071356	cellular response to tumor necrosis factor	1.02 × 10^−5^
GO:0014070	response to organic cyclic compound	1.168 × 10^−5^
GO:0048661	positive regulation of smooth muscle cell proliferation	1.242 × 10^−5^
GO:0071347	cellular response to interleukin‐1	1.37 × 10^−5^
GO:0009612	response to mechanical stimulus	1.508 × 10^−5^
GO:0045600	positive regulation of fat cell differentiation	2.168 × 10^−5^
GO:0043066	negative regulation of apoptotic process	2.473 × 10^−5^
GO:0033138	positive regulation of peptidyl‐serine phosphorylation	3.34 × 10^−5^
GO:0007249	I‐kappaB kinase/NF‐kappaB signaling	3.948 × 10^−5^
GO:0006955	immune response	4.057 × 10^−5^
GO:0045944	positive regulation of transcription from RNA polymerase II promoter	4.431 × 10^−5^
GO:0070373	negative regulation of ERK1 and ERK2 cascade	4.914 × 10^−5^
GO:0043123	positive regulation of I‐kappaB kinase/NF‐kappaB signaling	4.956 × 10^−5^
GO:0060546	negative regulation of necroptotic process	5.314 × 10^−5^
GO:2000116	regulation of cysteine‐type endopeptidase activity	7.62 × 10^−5^
GO:0002246	wound healing involved in inflammatory response	7.62 × 10^−5^
GO:0039535	regulation of RIG‐I signaling pathway	7.62 × 10^−5^
GO:0036018	cellular response to erythropoietin	7.62 × 10^−5^
GO:0070427	nucleotide‐binding oligomerization domain containing 1 signaling pathway	7.62 × 10^−5^
GO:0002237	response to molecule of bacterial origin	0.000103
GO:0051403	stress‐activated MAPK cascade	0.0001277
GO:0045651	positive regulation of macrophage differentiation	0.0001365
GO:0070555	response to interleukin‐1	0.0001673
GO:0010243	response to organonitrogen compound	0.0001673
GO:0032743	positive regulation of interleukin‐2 production	0.0001763
GO:0006953	acute‐phase response	0.0002129

We specifically scored for innervation, angiogenesis, and cell senescence, which are important events related to IVDD,^[^ ^19^ ^]^ based on GO terms and published literature (see the Experimental Section). It was suggested that there is no significant difference for innervation among NPC subpopulations (Figure 2D). For angiogenesis, fibroNPCs showed the highest score, and homeostatic NPCs showed the lowest score. (Figure 2E) Via SCENIC analysis, we identified the specific signature transcription factor motifs for each subpopulation, and their predictive transcriptional control (Figure S2B, Supporting Information).

Also, it was indicated that senescence‐associated secretory phenotype (SASP) was almost not expressed in HT‐CLNPs or homeostatic NPCs. However, HT‐CLNPs were active for cell cycle arrest (DNA damage) and SASP‐related epigenetics changes. FibroNPCs were highly active for macromolecular damage, indicating that HT‐CLNPs showed different cellular senescence mechanisms to FNPs (Figure 2F).

Overall, our findings suggest differences in functions and biological processes among NPC subpopulations. Moreover, multiple potential mechanisms for cell senescence exist in different subpopulations.

Two transcriptionally distinct clusters were identified: HT‐CLNP‐I and HT‐CLNP‐II. The DEGs of these two clusters are shown in Figure 2G. The HT‐CLNP‐I was enriched in genes related to programmed cell death and negative regulation of cell proliferation. In contrast, the HT‐CLNP‐II subset was enriched in genes related to ECM organization and ECM disassembly (**Table** **4**). Additionally, the Monocle pseudotime trajectory revealed progression of the HT‐CLNP‐I and HT‐CLNP‐II clusters (Figure 2H). These findings contribute to a deeper understanding of the hydrophilic mechanisms in NPCs during IVDD pathogenesis.

**Table 4:** Comparison of HT‐CLNPs‐I and HT‐CLNPs‐II

HT‐CLNPs‐I GO terms		
GOID	GOTerm	*P*‐value
GO:0006048	UDP‐N‐acetylglucosamine biosynthetic process	3.524 × 10^−7^
GO:0012501	programmed cell death	1.952 × 10^−6^
GO:0008285	negative regulation of cell proliferation	2.502 × 10^−6^
GO:0042026	protein refolding	3.032 × 10^−6^
GO:0010467	gene expression	1.394 × 10^−5^
GO:0043066	negative regulation of apoptotic process	1.964 × 10^−5^
GO:0031397	negative regulation of protein ubiquitination	2.025 × 10^−5^
GO:0010941	regulation of cell death	2.151 × 10^−5^
GO:0007596	blood coagulation	2.385 × 10^−5^
GO:1900740	positive regulation of protein insertion into mitochondrial membrane involved in apoptotic signaling pathway	4.689 × 10^−5^
GO:0045648	positive regulation of erythrocyte differentiation	4.689 × 10^−5^
GO:0002042	cell migration involved in sprouting angiogenesis	6.276 × 10^−5^
GO:1900034	regulation of cellular response to heat	7.543 × 10^−5^
GO:0006047	UDP‐N‐acetylglucosamine metabolic process	8.473 × 10^−5^
GO:0048010	vascular endothelial growth factor receptor signaling pathway	9.284 × 10^−5^
GO:0045944	positive regulation of transcription from RNA polymerase II promoter	0.0001026
GO:0006928	cellular component movement	0.0001158
GO:0006915	apoptotic process	0.0001216
GO:0034605	cellular response to heat	0.0001572
GO:0007623	circadian rhythm	0.0001957
GO:0032092	positive regulation of protein binding	0.0002012
GO:0042267	natural killer cell mediated cytotoxicity	0.0002278
GO:0034629	cellular protein complex localization	0.0002315
GO:0033173	calcineurin‐NFAT signaling cascade	0.0002315
GO:0045893	positive regulation of transcription, DNA‐templated	0.0002562
GO:0007264	small GTPase mediated signal transduction	0.0002668
GO:0006986	response to unfolded protein	0.0003037
GO:0032060	bleb assembly	0.000343
GO:0006357	regulation of transcription from RNA polymerase II promoter	0.0003793
GO:0097193	intrinsic apoptotic signaling pathway	0.0004046
GO:0006367	transcription initiation from RNA polymerase II promoter	0.0004339
GO:0050821	protein stabilization	0.0004345
GO:0010628	positive regulation of gene expression	0.0004551
GO:0061045	negative regulation of wound healing	0.0004841
GO:0070374	positive regulation of ERK1 and ERK2 cascade	0.000569
GO:0051085	chaperone mediated protein folding requiring cofactor	0.0006576
GO:0006366	transcription from RNA polymerase II promoter	0.0006606
GO:0045597	positive regulation of cell differentiation	0.0007432
GO:0060128	corticotropin hormone secreting cell differentiation	0.0007994
GO:0070370	cellular heat acclimation	0.0007994
GO:2000544	regulation of endothelial cell chemotaxis to fibroblast growth factor	0.0007994
GO:0043123	positive regulation of I‐kappaB kinase/NF‐kappaB signaling	0.0008046
GO:0033138	positive regulation of peptidyl‐serine phosphorylation	0.0010132
GO:0060129	thyroid‐stimulating hormone‐secreting cell differentiation	0.0015814
GO:0060591	chondroblast differentiation	0.0015814
GO:0010664	negative regulation of striated muscle cell apoptotic process	0.0015814
GO:0022614	membrane to membrane docking	0.0015814
GO:0015936	coenzyme A metabolic process	0.0015814
GO:0070434	positive regulation of nucleotide‐binding oligomerization domain containing 2 signaling pathway	0.0015814
GO:0045765	regulation of angiogenesis	0.0015841

HT‐CLNP‐II GO terms		
GOID	GOTerm	*P*‐value
GO:0030198	extracellular matrix organization	8.868 × 10^−15^
GO:0022617	extracellular matrix disassembly	9.881 × 10^−15^
GO:0006614	SRP‐dependent cotranslational protein targeting to membrane	1.733 × 10^−14^
GO:0006414	translational elongation	1.073 × 10^−13^
GO:0030574	collagen catabolic process	1.158 × 10^−13^
GO:0006415	translational termination	5.037 × 10^−13^
GO:0071294	cellular response to zinc ion	7.805 × 10^−13^
GO:0019083	viral transcription	7.948 × 10^−12^
GO:0000184	nuclear‐transcribed mRNA catabolic process, nonsense‐mediated decay	2.552 × 10^−11^
GO:0019058	viral life cycle	2.9 × 10^−10^
GO:0045926	negative regulation of growth	3.204 × 10^−10^
GO:0006413	translational initiation	8.987 × 10^−10^
GO:0016071	mRNA metabolic process	9.684 × 10^−10^
GO:0030199	collagen fibril organization	2.477 × 10^−9^
GO:0016070	RNA metabolic process	4.614 × 10^−9^
GO:0071276	cellular response to cadmium ion	5.567 × 10^−9^
GO:0001501	skeletal system development	2.605 × 10^−8^
GO:0006412	translation	4.843 × 10^−8^
GO:0016032	viral process	2.739 × 10^−7^
GO:0070208	protein heterotrimerization	1.033 × 10^−6^
GO:0009612	response to mechanical stimulus	1.974 × 10^−6^
GO:0071230	cellular response to amino acid stimulus	3.416 × 10^−6^
GO:0007568	aging	3.571 × 10^−6^
GO:0044267	cellular protein metabolic process	3.72 × 10^−6^
GO:0001957	intramembranous ossification	1.753 × 10^−5^
GO:0010467	gene expression	2.154 × 10^−5^
GO:0030168	platelet activation	2.648 × 10^−5^
GO:0007179	transforming growth factor beta receptor signaling pathway	3.104 × 10^−5^
GO:0009629	response to gravity	4.839 × 10^−5^
GO:0007566	embryo implantation	5.748 × 10^−5^
GO:0036018	cellular response to erythropoietin	9.356 × 10^−5^
GO:1903225	negative regulation of endodermal cell differentiation	9.356 × 10^−5^
GO:0035989	tendon development	9.356 × 10^−5^
GO:0043206	extracellular fibril organization	0.0001396
GO:0043065	positive regulation of apoptotic process	0.0001475
GO:0060707	trophoblast giant cell differentiation	0.0001848
GO:0043392	negative regulation of DNA binding	0.0002478
GO:0000302	response to reactive oxygen species	0.0002478
GO:0032570	response to progesterone	0.0002799
GO:0034097	response to cytokine	0.0002857
GO:0001666	response to hypoxia	0.0003199
GO:0007623	circadian rhythm	0.0003605
GO:0008284	positive regulation of cell proliferation	0.0005321
GO:0048705	skeletal system morphogenesis	0.0005377
GO:0048592	eye morphogenesis	0.0005542
GO:0097084	vascular smooth muscle cell development	0.0005542
GO:0032374	regulation of cholesterol transport	0.0005542
GO:0008283	cell proliferation	0.0006918
GO:0007155	cell adhesion	0.0007773
GO:0006898	receptor‐mediated endocytosis	0.0008835

### 2.4 CD90+NPCs Is the Progenitor within FibroNPCs, the End‐Stage Subpopulation

**Figure 3:**
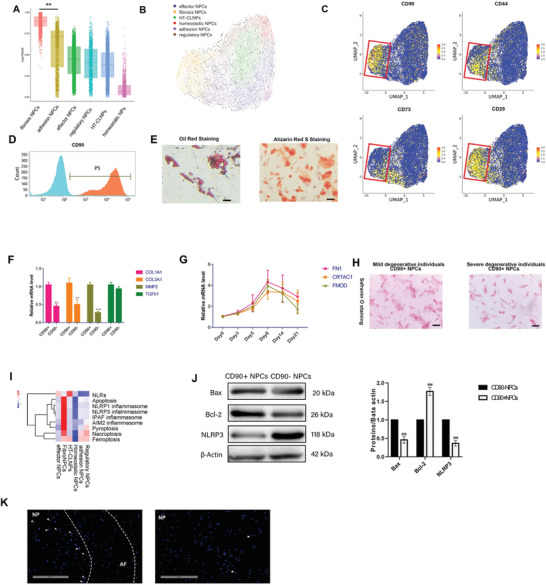
CD90+NPCs is the progenitor within FibroNPCs, the end‐stage subpopulation. A) Plot of the cytoTRACE pseudotime order for the NP subpopulations. The value of cytoTRACE represents the predicted order. B) Visualization for dynamic velocities projected into the UMAP‐based embedding. C) The expression of CD90, CD44, CD73, and CD29 in NPCs, the red box represents the region of fibroNPCs on UMAP. D) Histogram to evaluate the relative expression of CD90 after cell sorting. E) Left: oil red staining for CD90+ NPC‐induced adipogenic differentiation, respectively (*n* = 3). Scale bar, 100 µm. Right: alizarin red staining for CD90+ NPC‐induced osteogenic differentiation (*n* = 3). Scale bar, 100 µm. F) RT‐qPCR of fibroNPCs phenotype mRNA levels between CD90+/− NPCs. (*n* = 3 with mean ± SD shown, *P < 0.05, **P < 0.005). G) RT‐qPCR of adhesion NPCs phenotype at different time points after cultured CD90+NPCs in chondrogenesis induced medium (*n* = 3 with mean ± SD shown). H) Safranin O staining of CD90+NPCs from mild (grade II and III) or severely (grade IV and V) degenerative individuals after culturing in chondrogenesis medium for 21 d. I) Correlation of scRNA‐seq defined NPC subpopulations with cell death and inflammasome. J) Western blot analysis with representative blots including Bax, Bcl‐2m NLRP3 levels in the CD90+/− NPCs. Densitometric analysis is shown as mean ± SD, *n* = 3; *P < 0.05, **P < 0.005. K) Immunofluorescence (IF) visualization of CD90 (red) and nuclei (blue) in degenerative disc tissues induced in rats.

Next, we investigate the relationship between the different cell clusters. Cellular (Cyto) Trajectory Reconstruction Analysis using gene Counts and Expression (CytoTRACE) can accurately uncover the direction of differentiation and predict cell lineage trajectories.^[^ ^20^ ^]^ The results indicated the order of the differentiation states as fibroNPCs, adhesion NPCs, effector NPCs, regulatory NPCs, HT‐CLNPs, and homeostatic NPCs (**Figure** **3**A).

In addition, we applied the scVelo trajectory algorithms, a novel method developed for recovering the position of each cell in the underlying differentiation processed based on inferred gene‐specific rates of transcription, splicing, and degradation.^[^ ^21^ ^]^ The arrows in scVelo indicate the estimated sequence of transcriptomic events (Figure 3B). The results predict that the fibrosis NPCs may be differentiated into adhesion NPCs and other NPCs subsets. Monocle analysis also revealed that FNPs existed at the start of the pseudospace trajectory. Adhesion NPCs were distributed along the trajectory, and homeostatic NPCs were mainly distributed at the end (Figure S3A, Supporting Information). Together, these findings imply that fibroNPCs possess progenitor properties.

The scVelo analysis helped us to systematically identify putative driver genes as genes characterized by high likelihoods in fibroNPC populations (Figure S3B, Supporting Information). In other words, these genes may work as candidates for important drivers of the main process in firboNPCs. These genes have been associated with matrix remodeling (CoL14A1, coL12a1, CHAD, CRTAC1, TNC, Lamb1),^[^ ^22^ ^]^ antioxidation (FTH1),^[^ ^23^ ^]^ and inflammation (S100a8).^[^ ^24^ ^]^

Because fibroNPCs may have progenitor properties, we analyzed the expression of markers of mesenchymal stem cells (MSCs) in our single‐cell data and gated the fibroNPC region with MSCs markers (red box in Figure 3C) on visualized UMAPs. The results show that CD90 was expressed mainly in the fibroNPC region compared to the other MSC markers, CD44, CD73, and CD29. We, therefore, hypothesize that CD90+ NPCs may be the progenitor cells in degenerative NP tissues.

To confirm whether CD90+ NPCs are the progenitor cells in NP, we isolated CD90+NPCs from human degenerated NPCs using microbeads (Figure 3D). The CD90+NPCs were positive for CD44 and CD29 and negative for CD34 and HLA‐DR (Figure S5A, Supporting Information). Oil red staining and Alizarin red staining revealed that CD90+ cells had multipotent capabilities (differentiated into various cell lineages, including osteoblasts and adipocytes) (Figure 3E). RT‐qPCR showed that fibroNPCs phenotype genes (including CLO1A1, COL3A1, MMP2) were significantly elevated in CD90+NPCs compared to CD90‐NPCs, however, the expression of TGFb *β* showed no significant difference between two groups (Figure 3F). We also cultured CD90+NPCs in the chondrogenesis differentiation medium and found that adhesion NPCs genes, including FN1, CRTAC1, and FMOD, were elevated from day 1 and peaked at day 6 but decreased after that (Figure 3G). This result indicates that CD90+NPCs can differentiate into cells with adhesion NPCs phenotypes. Safranin O staining showed a higher chondrogenesis potential for CD90+ NPCs isolated from mild degenerative (grade II and III) individuals compared to those from severe degenerative (grade IV and V)individuals (Figure 3H). These findings demonstrate that CD90+NPCs expressed phenotypes of fibroNPCs and can serve as progenitor cells in degenerative NP tissues.

Both cell repair and cell death are involved in tissue regeneration.^[^ ^25^ ^]^ To explore whether different cell death events occurred in our predictive NPC subsets, we correlated our single‐cell data with previous publications.^[^ ^26^ ^]^ The correlation analysis showed that fibroNPCs were active for all four cell death types: apoptosis, pyroptosis, necroptosis, and ferroptosis. Inflammasome may be highly involved in cell death in fibroNPCs but not in other cell types. Ferroptosis was evident in HT‐CLNPs, adhesion NPCs, and regulatory NPCs. Necroptosis occurred in fibroNPCs, adhesion NPCs, and regulatory NPCs (Figure 3I). Next, we compared apoptosis and NLRP3‐related proteins expressed between CD90+ NPCs and CD90− NPCs; CD90+ cells showed decreased levels of the Bax and NLRP3 transcripts, as well as increased Bcl‐2 expression (Figure 3J). Furthermore, we used immunofluorescence to explore the location of CD90+ NPCs in puncture‐induced degenerative rat IVD.^[^ ^3^ ^]^ It was found that CD90+ NPCs were enriched in the boundary between NP and AF; only a few positive cells could be detected in the inner area of the discs (Figure 3K). Therefore, the combined results showing fibroNPCs showed profound cell death activity. Nevertheless, CD90+NPCs may help NP regeneration in this subpopulation. As it was shown in Figure 1D, the proportion of fibroNPCs is elevated with degeneration progression, which indicating fibroNPCs could be the end‐stage subpopulation. Overall, fibroNPCs are the major cell population for end‐stage degenerative NPCs with the persistence of both cell death and progenitors. The CD90+NPCs as the progenitor NPCs can be a potential therapeutic target in the future study.

### 2.5 Identification of NP‐Derived G‐MDSCs

**Figure 4:**
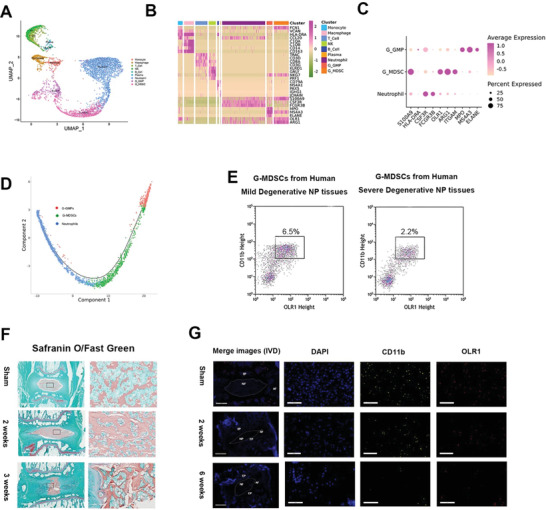
Identification of NP‐derived G‐MDSCs. A) Uniform manifold approximation and projection (UMAP) visualization showing immune cells inside NP tissues. B) Heatmap showing the typically expressed genes in each cell type. C) Dot plot showing scaled expression of selected signature genes for GMP, G‐MDSCs, and neutrophils, by average expression of each gene in each cluster scaled across all clusters. Dot size represents the percentage of cells in each cluster with more than one read of the corresponding gene. D) Monocle method reconstructed pseudospace trajectory for GMP, G‐MDSC, and neutrophils. E) FACS isolation for CD45+ CD11b+OLR+ cells from mild/severe degenerative NP tisues. F) Safranin O/Fast Green staining of the intervertebral discs sham and experimental rats. Scale bar, 1 mm in left and 100 µm in right. G) Merged immunofluorescence staining of DAPI, CD11b, OLR1 in the intervertebral discs of sham and experimental rat. Scale bar, 1 mm in merge images and 100 µm in others.

Apart from NPCs, immune cells were identified in NP tissues. Using the canonical correlation analysis (CCA) method,^[^ ^27^ ^]^ natural killer (NK) cells were identified by CD94.^[^ ^28^ ^]^ Macrophage/monocytes were identified by CD163,^[^ ^29^ ^]^ and T cells were identified by T Cell Receptor Alpha Constant (TRAC) (Figure 1B,C). G‐GMPs (expressing MS4A3, MPO, and ELANE),^[^ ^30^ ^]^ neutrophils (FCGR3B (CD16b) + HLADR),^[^ ^31^ ^]^ and granulocytic myeloid‐derived suppressor cell (G‐MDSC, ITGAM (CD11b), OLR1, and ARG1)^[^ ^32^ ^]^ were also identified (**Figure** **4**A–C). Monocle trajectory analysis revealed GMPs were distributed at the start of the trajectory, G‐MDSCs at the intermediate segment, and neutrophils were dispersed along the trajectory (Figure 4D).

Our data show that there is an enrichment of G‐MDSCs in severely degenerated NP tissues (grade IV and V) compared to mildly degenerative tissues (grade II and III) at the single‐cell level. To confirm this finding, we isolated G‐MDSCs from human NP tissues via Fluorescence‐activated cell sorting (FACS) (Figure S4A, Supporting Information). CD45, the marker of hematopoietic cells, CD11b, OLR1 were markers used for G‐MDSCs sorting. G‐MDSCs (CD45+CD11b+OLR1+ cells) decreased by almost threefolds in severe degeneration discs (6.5% vs 2.2%) (Figure 4E). To further validate this finding in vivo, we used rat models of IVDD with the needle puncture method.^[^ ^3^ ^]^ Histological staining showed notable changes including cell cloning (cell clusters), loss of demarcation between NP and AF, and proteoglycan loss in punctured IVDs, demonstrating progressive IVDD at two and six weeks (Figure 4F). Immunofluorescence staining for CD11b, OLR, and CD24 showed the existence of G‐MDSCs in rat NPs, and G‐MDSCs decreased in the six‐week group relative to the two‐week group (Figure 4G).

### 2.6 Functional Validation NP‐Derived MDSCs

**Figure 5:**
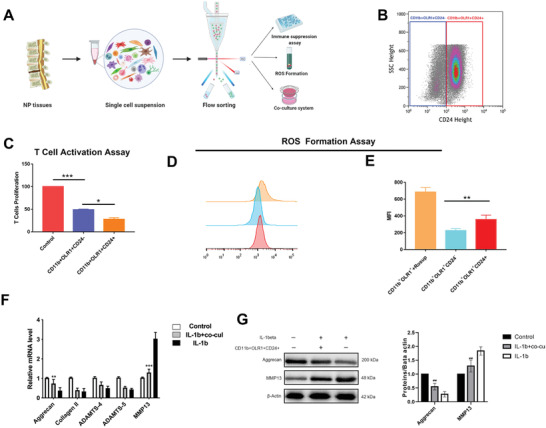
Validation of functions of NP‐derived G‐MDSCs. A) Schematic workflow of the experimental strategy. B) FACS isolation for CD45+ CD11b+OLR+CD24+ and CD24− cells. C) T cell suppression analysis NP‐derived G‐MDSC identification. *N* = 3, *P < 0.05, **P < 0.005, ***P <0 .001. D,E) CD11b+OLR+CD24+ show increased reactive oxygen species (ROS) formation compared to CD24− cells. Rosup treated cells were used as positive control. *N* = 3, *P < 0.05, **P < 0.005. F) RT‐qPCR of degeneration related genes, aggrecan, collagen II, ADAMTS4,5, and MMP13 in untreated, IL‐1b+G‐MDSCs, and IL‐1b alone NPC groups. *N* = 3, *P < 0.05, **P < 0.005, ***P < 0.001. G) Western blot analysis with representative blots including aggrecan and MMP13 in untreated, IL‐1b+G‐MDSCs, and IL‐1b alone NPC groups. *N* = 3, *P < 0.05, **P < 0.005, ***P < 0.001.

The most typical and important function of G‐MDSCs is immunosuppression.^[^ ^33^ ^]^ The hallmark of this immunosuppressive activity is the capability of suppressing T cell activation and ROS production.^[^ ^34^ ^]^ Next, we validate the functions of NP‐derived MDSCs via immunosuppression and ROS production (**Figure** **5**A).

As we found that CD24 also highly expressed in MDSCs at single‐cell level, we tested this finding by FACS. As it is shown in Figure 5B, the NP‐derived MDSCs were divided into CD24+ MDSCs and CD24‐MDSCs. To functionally validate whether CD45+CD11b+OLR1+CD24+/− cells inhibit immune cell activation, we performed co‐cultures with activated T cells (Figure 5C).^[^ ^35^ ^]^ Human NP tissues, both CD45+CD11b+OLR1+CD24+ and CD24−, exhibited T cell suppressive capacity; however, CD24+ cells showed significantly stronger suppressive capacity compared to CD24− cells (Figure 5C). Similar to T‐cell proliferation, interleukin 2 (IL2) production was significantly suppressed by G‐MDSC co‐cultured (Figure S4B, Supporting Information). We also observed that CD45+CD11b+OLR1+CD24+ cells produced significantly higher amounts of ROS compared to CD24− cells (Figure 5D,E). These findings indicate that our identified G‐MDSCs in human NP tissues have T‐cell suppression and ROS production capabilities.

Because G‐MDSCs decreased in severely degenerated NP tissues, we hypothesized that G‐MDSCs might protect against IVDD. To explore the effects of G‐MDSCs on NPCs, G‐MDSCs were isolated from mildly degenerated (grade II and III) human NP tissues and co‐cultured with non‐degenerative NPCs. IL‐1*β* was used to induce NPC degeneration.^[^ ^36^ ^]^ Consequently, compared with IL‐1*β* treatment alone, G‐MDSCs co‐cultured with NPCs showed increased aggrecan expression and significantly lower MMP13 expression (Figure 5F,G). These in vitro findings indicate that G‐MDSCs could alleviate IL‐1*β* induced NPCs matrix degradation.

### 2.7 Characterization of Cell‐to‐Cell Interactions Involved in IVDD

**Figure 6:**
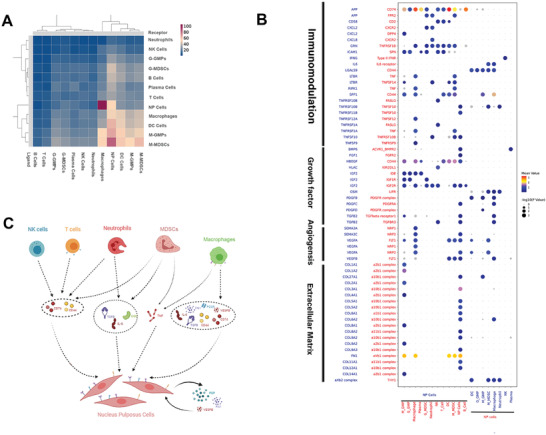
Predicted immune‐NPCs regulatory network in IVDD. A) Heatmap showing the number of potential ligand–receptor pairs between cell groups. B) Bubble plots showing ligand–receptor pairs of immunomodulation, growth factors, angiogenesis, and adhesion between NPCs and other cell groups. C) Predicted regulatory network centered on NPCs.

Our study showed that NP tissues consist of NPCs and immune cells. We next predicted the cell–cell interaction network among the cell types using CellPhoneDB 2.0.^[^ ^37^ ^]^ Macrophages showed the most interactions with other cell types. Interactions among NPCs were the most active (**Figure** **6**A).

CD74/APP and CD44/HBEGF widely existed in the interaction between immune cells and NPCs. NPCs also produced VEGFB and VEGFA, which bind to VEGF receptors (FLT1, ADRB2, NRP1, and NRP2) in both NPCs and immune cells, especially macrophages. This implies that the interaction between macrophages and NPCs is potentially proangiogenic. Notably, a healthy IVD is largely avascular. However, angiogenesis occurs during degeneration.^[^ ^38^ ^]^

It has also been suggested that NPCs produce FN1 and FGF for their receptors. Immunomodulation‐related cytokines, such as pro‐inflammatory cytokine, TNF, anti‐inflammatory cytokines, and IL‐6, are expressed by macrophages, neutrophils, and G‐MDSCs, and can bind to NPC receptors. Neutrophils and macrophages expressed growth factors, including TGFB, PDGF, and OSM, and showed pro‐proliferation effects on NPCs (Figure 6B,C). Overall, our results predicted a network between immune cells and degenerative NPCs, which may play a role in inflammation balance, cell proliferation, and angiogenesis in NP. However, the exact mechanisms should be confirmed by further experiments.

## 3 Discussion

Our identified subpopulations showed transcriptomic heterogeneity and potential functional differences at the single‐cell level. Effector NPCs were identified and indicated to be active in cellular anabolism and catabolism activities, as they showed a high metabolic rate, including sterol biosynthesis, glycosaminoglycan metabolism, and protein metabolism. During IVDD, the ECM and cells undergo profound metabolic processes.^[^ ^39^ ^]^ The QuSAGE analysis indicated effector NPCs are active for “positive regulation of ECM assembly,” which is opposite to fibroNPCs with “negative regulation of ECM assembly.” Thus, effector NPCs may play a role in maintaining ECM homeostasis. It was also suggested that with IVDD progressing, the proportion of effector NPCs decreased, while fibroNPCs proportion increased. Regulatory NPCs are characterized by inflammation and endogenous stimuli responses. The Toll‐like signaling pathway, a crucial pathway related to the innate immune system, is active in regulatory NPCs. Moreover, regulatory NPCs have been shown to be one of the main subsets in grade II NP degeneration, suggesting that they may serve as a cell population for immunity maintenance. Calió et al. found 15 distinct cell clusters in healthy bovine NP tissues. Their study demonstrated that NP is heterogeneous, they also identified notochordal‐like cell cluster and a progenitor stem cell cluster inside the bovine NP.^[^ ^40^ ^]^

During cartilage development, chondrocytes undergo terminal differentiation when they become hypertrophic. It has been widely acknowledged that chondrocyte hypertrophy‐like changes play a role in osteoarthritis (OA).^[^ ^41^ ^]^ It has been shown that hypertrophic differentiation occurs in NP tissues during IVDD progression.^[^ ^42^ ^]^ Here, we annotated a cell population as “HT‐CLNPs” and identified HT‐CLNPs‐I and HT‐CLNPs‐II subpopulations possessing different ontologies. The HT‐CLNP‐I subpopulation was enriched in genes related to negative regulation of apoptosis, response to unfolded protein, and circadian rhythm, while the HT‐CLNP‐II subpopulation expressed genes involved in ECM organization and disassembly (Table 3). This indicates that HT‐CLNP‐I may serve as a regulatory cell cluster that may play a potential protective role in chondrocyte hypertrophy‐like events. In support, our data showed decreased HT‐CLNP‐II proportion in mild degenerative discs (grade II and III). Moreover, a distinct cell marker of HT‐CLNP‐II, CHRDL2, was found to negatively regulate chondrocyte development by competitively inhibiting BMPs.^[^ ^43^ ^]^ BMP/TGF*β* is a vital pathway promoting chondrocyte hypertrophy. Here, functional analysis (Figure 3I) showed different cell death events occurring in HT‐CLNPs compared to other subpopulations. In HT‐CLNPs, ferroptosis, instead of apoptosis, was the main cell death type. HT‐CLNPs also showed decreased cell senescence and a low level of SASP. These results revealed potential cell dysfunction events happened in NPCs chondrocyte hypertrophy‐like changes.

It has been reported that NP originates from the notochord; however, at about age 4 in humans, notochordal cells disappear, replaced by smaller non‐vacuolated cells. More importantly, the origin of these cells is unknown. Previous studies indicated that Tie2 positive (Tie2+) and disialoganglioside 2 positive (GD2+) populations serve as progenitor NPCs.^[^ ^44^ ^]^ However, few Tie2+ and GD2+ NPCs can be found in patients aged over 50.^[^ ^45^ ^]^ Interestingly, the single‐cell data showed that there are few Tie2+ or GD2+ NPCs in degenerative NP tissues (Figure S5B, Supporting Information). IVDD is an age‐related condition; the average age is 59.13 ± 12.5 for males and 61.02 ± 10.6 for females.^[^ ^46^ ^]^ Thus, finding out progenitor cells in aged and degenerative NP tissues is vitally important for understanding tissue homeostasis and regeneration. Blanco et al. identified the presence of nucleus pulposus mesenchymal stem cells (NP‐MSCs) within the IVD. And their study showed the NP‐MSCs do not have adipogenic differentiation ability.^[^ ^47^ ^]^ Our scRNAseq showed that fibroNPCs demonstrated the highest score for progenitor properties and stemness. Increasing evidence shows that apoptotic cells can induce compensatory proliferation and promote regeneration in invertebrates and vertebrates.^[^ ^25^ ^]^ The relationship among apoptosis, proliferation, and tissue regeneration has been linked in some animal models, like drosophila, planarians, newts, and mice.^[^ ^48^ ^]^ Cell apoptosis is an important cell death mechanism in IVDD.^[^ ^19b^ ^]^ Our study showed that apoptosis and inflammasome activity occurred mainly in fibroNPC populations. Moreover, the score for angiogenesis is highest in fibroNPCs and angiogenesis is essential for the growth and regeneration of tissues.^[^ ^49^ ^]^ These imply that fibroNPCs are a significant subpopulation for cell regeneration in NP tissues.

We selected CD90+ NPCs as candidate progenitors, which transcriptionally co‐express genes for fibroNPCs and MSCs. CD90+NPCs showed decreased apoptosis compared to CD90‐ NPCs and could differentiate into adipocytes, osteoblasts, and other NPCs atlas (like adhesion NPCs). Furthermore, the CD90+NPCs isolated from mild degeneration individuals (grade II and III) demonstrated increased cell chondrogenesis ability. This study revealed that CD90+ is important for maintaining NP homeostasis and can be used in cell‐based scaffolding for NP repair and regeneration. Moreover, CD90+ was found most abundant in the NP/AF boundary regions in rat degenerative disc tissues. Exploring the origin of progenitor cells is of researchers’ interest. Previous descriptive studies found that progenitor and stem cell niche patterns in healthy IVDs are detected in certain AF areas. It was possible that MSCs from the bone marrow niche were involved in NP origination.^[^ ^50^ ^]^ Here, we hypothesized that a potential niche with CD90+NPCs enriched exists in the NP/AF boundary (Figure S5C, Supporting Information). CD90+NPCs may originate from the AF region, perichondrium region, or from bone marrow‐MSCs differentiation and migration. Detailed experiments should be conducted to trace the origin and differentiation of CD90+NPCs in the future.

The IVDs have been identified as immune privilege organs; the steady state of immune privilege is fundamental to organ homeostasis.^[^ ^51^ ^]^ Although the NP is often described in general terms in the literature as being avascular, previous studies have reported blood vessels can penetrate the NP, especially when degeneration. Binch et al. reported the “abnormal” localization of blood vessels within the NP associated with degeneration.^[^ ^52^ ^]^ Freemont et al. indicated the blood vessels occurs into normally aneural and avascular regions of the IVD, notably the NP in painful IVDD patients.^[^ ^53^ ^]^ Considering the angiogenesis might link to disturbed cell biology, we explored the correlation between angiogenesis and NPCs heterogeneity. We found that the angiogenesis scores are highest in firboNPCs, but lowest in homestatic NPCs (Figure 2E). We also founded the homeostatic NPCs showed negative correlations with degeneration severity and fibroNPCs showed positive correlations with severity (Figure 1D). The angiogenesis in NP is also associated with in‐growth of nerves, which contribute to chronic back pain symptoms.^[^ ^54^ ^]^ David et al. showed the process of angiogenesis at the IVD affects the patient's quality of life both pre and postoperatively.^[^ ^55^ ^]^ The angiogenetic NPCs populations might contribute to angiogenesis by express angiogenic factors like vascular endothelial growth factor (VEGF). The neovascularization can bring cytokines and immune cells. The endothelial cells of these blood vessels produce nerve growth factor (NGF) which previously has been shown to be responsible for the in‐growth of nociceptive nerve fibers into the normally aneural IVD.^[^ ^56^ ^]^ The angiogenetic NPCs populations therefore might accelerate degeneration and lead to chronic back pain. However, more experiments should be conducted to elucidate their significance in the future.

This single‐cell study showed there are multiple immune cell lines inside the NP, which may play a role in IVDD progression. Based on correlation analyses of previously reported single‐cell data, we showed that G‐GMPs aligned with bone marrow GMPs, proNeu, and preNeu. G‐MDSCs aligned with CXCR2^low^ immNeu and CXCR2^high^ mNeu, and neutrophils aligned with polymorphonuclear leukocytes (PMNs).^[^ ^57^ ^]^ G‐MDSC represents a group of immature broadly defined neutrophils with immunosuppressive functions characterized as CD14−/CD11b+/CD15+/CD66b+ in human.^[^ ^58^ ^]^ MDSCs have been widely studied in cancer, acting as a suppressor of antitumor immune responses.^[^ ^59^ ^]^ Accumulation of G‐MDSCs has also been reported in cancers and other inflammatory diseases, including inflammatory bowel diseases, rheumatoid arthritis, autoimmune arthritis, and autoimmune hepatitis.^[^ ^60^ ^]^ Nevertheless, the involvement of MDSCs in IVDD has not been revealed. MDSCs have a close correlation with neutrophils. Our findings indicated that in NP tissues, G‐MDSCs might emerge from GMPs through a differentiation trajectory.

Although we identified CD24, which can be used in combination with CD11b and OLR1 to detect the presence of G‐MDSCs in degenerative NP tissues, this finding does not deny the CD24 as a marker for NPCs. The expression of CD24 is decreased with IVDD severity.^[^ ^61^ ^]^ Previously, CD24+ NPCs were identified as progenitors in NP.^[^ ^62^ ^]^ In humans, the percentage of CD24+NPCs decreased significantly with aging or degeneration, which dropped to less than 10% in elderly adults or severely degenerated discs.^[^ ^63^ ^]^ In our study, scRNAseq showed that there are few CD24+ NPCs in degenerative discs. That is because there is only one participant below 50 years old in this study. Here, we identified CD24 as a cell marker for detecting NP‐derived G‐MDSCs when combined with CD11b and OLR1.^[^ ^32^, ^59^ ^]^ The results of FACS showed that most CD11b+OLR1+ were CD24 positive. Thus, we can conclude that CD24 is mainly expressed in G‐MDSCs, but not in NPCs, especially in elderly populations. The immunofluorescence for CD24 in discs of rat models displayed positivity for NPCs, and the percentage of positive cells decreased with IVDD.^[^ ^64^ ^]^This might be a result of age and species difference as the rats we used were 2–3 months old. It is currently known that CD24 serves as a costimulatory factor of T cells, regulating their homeostasis and proliferation, and is involved in B cell activation and differentiation.^[^ ^65^ ^]^ Our findings showed CD24 can regulate suppression of T cell responses, suggesting CD24 might allow G‐MDSCs to directly regulate immune checkpoints in NP tissues. Our single‐cell data and animal experiments showed that G‐MDSCs substantially decreased in mildly degenerated NP tissues. Our co‐culture experiment also suggested G‐MDSCs might be a potential treatment option for IVDD.

Although our study provides some previously unrevealed insights into NP tissue biology, there are potential limitations to our study here. First, we need to acknowledge that part of the functional analysis was based on scRNAseq prediction. For instance, the functional difference was among six identified NPC subsets. Other alternative methods should be used to estimate the functional biology behind transcriptional changes in future research. Second, the dissociation bias when processing tissues for scRNAseq may lead to spurious changes of cell distribution, which can limit our ability to provide an exact numeric description of cell population changes. Several strategies were undertaken to minimize the bias. First, all samples were handled following the same protocol. Second, sample acquisition was carefully made during the surgery to avoid contamination of AF tissues or other tissues. Third, we used multiple methods to show the presence of identified novel cell types, like G‐MDSCs. In summary, we have comprehensively decoded the multicellular ecosystem of NP during degeneration. These findings may be leveraged to improve diagnostics and develop preventative strategies for degenerative spinal diseases.

## 4 Experimental Section

### NP Tissues Specimens

This study protocol was approved by the Ethics Committee of the First Affiliated hospital of Soochow University. All participants signed a written informed consent form. The enrolled subjects were patients who required discectomy and/or interbody fusion for burst fracture (*n* = 2) lumbar disc herniation or spondylolysis (*n* = 6). The patients’ characteristics were listed in Table 1. In this study, the grading of IVDD based on Pfirrmann grading system. It should be noted that it is difficult to obtain Pfirrmann grade I disc tissues, especially in elderly participants. Thus, only grade II to V discs were analyzed in this study (Figure S1A, Supporting Information).

A standard surgical protocol assured correct sample acquisition and thus accurate selection of AF and NP tissue by experienced surgeons. For burst fracture cases, the NP tissue from the disc attached to the intact bottom endplate was harvested. For severe degenerative discs, the tissue from the central region of the NP was harvested to assure no AF tissues were harvested. If local bleeding happened during the tissue harvested, the tissues were discarded to ensure no contaminated blood in collected tissues.

Tissues immediately following surgical procurement were processed, and single‐cell suspensions were generated within ≈45 min with an experimental protocol optimized to reduce artifactual transcriptional changes introduced by disaggregation, temperature, or time. Human NP tissue samples were transported in MACS Tissue Storage Solution (Miltenyi Biotec) immediately after surgical resection. After tissues were harvested, the specimens were washed with PBS for three times and further checked under a dissecting microscope to guarantee there was no contamination of blood, AF tissues, or other tissues. For few cases which were difficult to distinguish between the NP and inner AF, histological staining was used to confirm the absence of AF tissues or other tissues (Figure S1B–D, Supporting Information).

### Single‐Cell Dissociation of Human Nucleus Pulposus

The tissues were surgically removed and kept in MACS Tissue Storage Solution (Miltenyi Biotec) until processing. The tissue samples were processed as described below. Briefly, samples were first washed with phosphate‐buffered saline (PBS), minced into small pieces (≈1 mm^3^) on ice and enzymatically digested with 500 U mL^−1^ collagenase I, 150 U mL^−1^ collagenase II, 50 U mL^−1^ collagenase IV, 0.1 mg mL^−1^ hyaluronidase, 30 U mL^−1^ DNaseI, and 5% Fetal Bovine Serum Oringin South America (Yeasen) for 95 min at 37 °C, with agitation. After digestion, samples were sieved through a 70 µm cell strainer, and centrifuged at 300×*g* for 5 min. After washing with PBS containing 0.04% BSA, the cell pellets were re‐suspended in PBS containing 0.04% BSA and re‐filtered through a 35 µm cell strainer. Dissociated single cells were then stained with AO/PI for viability assessment using Countstar Fluorescence Cell Analyzer. The single‐cell suspension was further enriched with a MACS dead cell removal kit (Miltenyi Biotec).

### Single‐Cell RNA Sequencing

BD Rhapsody system was used to capture the transcriptomic information of the (8 sample‐derived) single cells. Single‐cell capture was achieved by random distribution of a single‐cell suspension across >200 000 microwells through a limited dilution approach. Beads with oligonucleotide barcodes were added to saturation so that a bead was paired with a cell in a microwell. Cell lysis buffer was added so that poly‐adenylated RNA molecules hybridized to the beads. Beads were collected into a single tube for reverse transcription. Upon cDNA synthesis, each cDNA molecule was tagged on the 5′ end (i.e., the 3′ end of an mRNA transcript) with a unique molecular identifier (UMI) and cell label indicating its cell of origin. Whole transcriptome libraries were prepared using the BD Rhapsody single‐cell whole transcriptome amplification workflow. In brief, second strand cDNA was synthesized, followed by ligation of the WTA adaptor for universal amplification. Eighteen cycles of PCR were used to amplify the adaptor‐ligated cDNA products. Sequencing libraries were prepared using random priming PCR of the whole‐transcriptome amplification products to enrich the 3′ end of the transcripts linked with the cell label and UMI. Sequencing libraries were quantified using a High Sensitivity DNA chip (Agilent) on a Bioanalyzer 2200 and the Qubit High Sensitivity DNA assay (Thermo Fisher Scientific). The library for each sample was sequenced by illumina sequencer (Illumina, San Diego, CA) on a 150 bp paired‐end run.

### Single‐Cell RNA Statistical Analysis

fastp^[^ ^66^ ^]^ with default parameter was applied filtering the adaptor sequence, and the low quality reads were removed to achieve the clean data. UMI tools were applied for Single Cell Transcriptome Analysis to identify the cell barcode whitelist. The UMI‐based clean data were mapped to human genome (Ensemble version 91) using STAR mapping with customized parameter from UMI tools standard pipeline to obtain the UMIs counts of each sample. Cells contained over 200 expressed genes and mitochondria UMI rate below 20% passed the cell quality filtering and mitochondria genes were removed in the expression table. Seurat packageSeurat package (version: 2.3.4, https://satijalab.org/seurat/) was used for cell normalization and regression based on the expression table according to the UMI counts of each sample and percent of mitochondria rate to obtain the scaled data. To correct the batch effect, fastMNN function from scater package (https://github.com/Alanocallaghan/scater/tree/master/R) was applied with k value equals 5 and UMAP as well as tSNE dimension reduction construction was calculated following the batch effect correction result. PCA was constructed based on the scaled data with top 2000 high variable genes and top 10 principals were used for tSNE construction and UMAP construction. Using graph‐based cluster method (resolution = 0.8), the unsupervised cell cluster result based on the PCA top 10 principals acquired, and the marker genes were calculated by FindAllMarkers function with Wilcox rank sum test algorithm under following criteria: lnFC > 0.25; *p*‐value < 0.05; and min.pct > 0.1. To identify the cell type detail, the clusters of same cell type were selected for re‐tSNE analysis, graph‐based clustering, and marker analysis. To identify differentially expressed genes among samples, the function FindMarkers with Wilcox rank sum test algorithm was used under following criteria: lnFC > 0.25; *p* value < 0.05; and min.pct > 0.1.

### Pseudo‐Time Analysis

The single‐cell trajectories analysis was applied using Monocle2 (http://cole‐trapnell‐lab.github.io/monocle‐release) using DDR‐Tree and default parameter. Before Monocle analysis, marker genes of the Seurat clustering result and raw expression counts of the cell passed filtering were selected. Based on the pseudo‐time analysis, branch expression analysis modeling (BEAM Analysis) was applied for branch fate determined gene analysis.

More single‐cell trajectories analyses were applied by using PAGA in scanpy package (https://scanpy.readthedocs.io/en/latest/index.html, version 1.6.0) and Slingshot (https://bioconductor.org/packages/release/bioc/html/slingshot.html, version 1.4.0). Before analysis, marker genes of the Seurat clustering result and raw expression counts of the cell passed filtering were selected.

### Cell Communication Analysis and SCENIC Analysis

To enable a systematic analysis of cell–cell communication molecules, cell communication analysis based on the CellPhoneDB, a public repository of ligands, receptors, and their interactions, was applied. Membrane, secreted and peripheral proteins of the cluster of different time point were annotated. Significant mean and cell communication significance (*p*‐value < 0.05) was calculated based on the interaction and the normalized cell matrix achieved by Seurat Normalization. To assess transcription factor regulation strength, the single‐cell regulatory network inference and clustering (pySCENIC, v0.9.5) workflow was applied, using the 20 000 motifs database for RcisTarget and GRNboost.

### QuSAGE Analysis (Gene Enrichment Analysis)

To characterize the relative activation of a given gene set such as pathway activation, “CellDeath_Ferrdb_soring” and “CellDeath_Inflammasome_review” as described before, QuSAGE (2.16.1)^[^ ^18a^ ^]^ analysis.

### Cell Fate Analysis, Including CytoTRACE and Velocity

The scVelo package with default parameter was applied for studying the cellular differentiation status based on the bam mapping file from UMI tools STAR mapping steps to solve the transcriptional dynamics of splicing kinetics using a likelihood‐based dynamical model. CytoTRACE Analysis was applied for cell stem stage analysis with default parameter.^[^ ^20^ ^]^

### Pathway Analysis

Pathway analysis was used to find out the significant pathway of the marker genes and differentially expressed genes according to KEGG database. Fisher's exact test was used to select the significant pathway, and the threshold of significance was defined by *P*‐value and FDR.

### Rat Model of IVDD

Animal experiments were conducted following the guidelines of the Ethical Committee of The First Affiliated Hospital of Soochow University. A total of 15 male Sprague‐Dawley rats, aged three months, were used for the experiments in vivo. Ten rats underwent the surgeries, and the remaining five rats underwent no surgical intervention as negative controls. On the day of surgery, the animals were anesthetized in the induction chamber with oxygen flow at 1 L min^−1^ and up to 4% isoflurane. The isoflurane was reduced to between 2% and 2.5% once the animal was asleep. Once anesthetized, the animal was placed on a heating pad in supine position. Buprenorphine (0.1 mg kg^−1^) in saline were administered by subcutaneous injection for post operation pain relief. The intervertebral space was located by digital palpation. Next, a needle was affixed using a clamp so that 5 mm tip sticks out. After cleaned injection site with ethanol, the needle head was then inserted into the intervertebral space and held in place for 20 s to ensure puncture. After surgeries, the animals were returned to a warm and clean cage and monitored until wake.

### Quantitative Real‐Time PCR

The samples used for RT‐qPCR were independent sample pools from the sampled used for sequencing. The total RNA was harvested from the NPCs, and it was reverse transcribed into cDNA with SuperScript II reverse transcriptase (Invitrogen, Cat No. 18064014). The sequence of primers for RT‐PCR are as below: MSMO1 (Forward 5′‐3′: TGCTTTGGTTGTGCAGTCATT; Reverse 5′‐3′: GGATGTGCATATTCAGCTTCCA) HMGS1 (Forward primer: GAGCCCATACTCATCAAGTACCG; Reverse primer: CCTCGGGAGAGATGCACAC); DKK (Forward primer: ACGAGTGCATCATCGACGAG; Reverse primer: GCAGTCCCTCTGGTTGTCAC); FN1 (Forward primer: AGGAAGCCGAGGTTTTAACTG; Reverse primer: AGGACGCTCATAAGTGTCACC); CRTAC1 (Forward primer: TGTCCAGGATGTTACCGTTCC; Reverse primer: AGCTGGGTGGGATTACTGTCA); COL1A1 (Forward primer: ATCAACCGGAGGAATTTCCGT; Reverse primer: CACCAGGACGACCAGGTTTTC); COL3A1 (Forward primer: GGAGCTGGCTACTTCTCGC; Reverse primer: GGGAACATCCTCCTTCAACAG); MMP2 (Forward primer: CCCACTGCGGTTTTCTCGAAT; Reverse primer: CAAAGGGGTATCCATCGCCAT); RPS29 (Forward primer: CGCTCTTGTCGTGTCTGTTCA; Reverse primer: CCTTCGCGTACTGACGGAAA); RPS21 (Forward primer: AGCAATCGCATCATCGGTG; Reverse primer: CCCCGCAGATAGCATAAGTTTTA); CHI3L1 (Forward primer: AAGCAACGATCACATCGACAC; Reverse primer: TCAGGTTGGGGTTCCTGTTCT); NF‐kB (Forward primer: AACAGAGAGGATTTCGTTTCCG; Reverse primer: TTTGACCTGAGGGTAAGACTTCT); CXCL2 (Forward primer: CTTGTCTCAACCCCGCATC; Reverse primer: CAGGAACAGCCACCAATAAGC); FMOD (Forward primer: ATTGGTGGTTCCACTACCTCC; Reverse primer: GGTAAGGCTCGTAGGTCTCATA).

### Scoring of Biological Processes

Individual cells were scored for their expression of gene signatures representing certain biological functions. The functional signatures were derived from the Gene Ontology database. The immune response and steroid biosynthetic process were measured by GO:0006955 and GO:0006694, respectively. The innervation score was measured by the calculating the average expression of genes in the Gene Ontology term “innervation” (GO:0060384). The angiogenesis was measured by term “positive regulation of angiogenesis” (GO:0045766). The indicated scores were calculated by scaling the normalized expression of a gene across all cells. Gene weights were set to either 1 or −1 to reflect positive or negative relationships.

### Immunohistochemical (IHC) assays

The samples used for IHC were independent sample pools from the sampled used for sequencing. NP tissues were fixed for 48 h in 4% buffered paraformaldehyde. The sections were pre‐treated for 10 min with trypsin (0.05%) and then treated with 3% (vol/vol) H_2_O_2_ for 15 min. Then, the sections were blocked at room temperature for 1 h with 10% goat serum. After washing with PBS, sections were incubated with anti‐CRTAC1 (1:50 dilution, ab25469, Abcam), anti‐CHI3L1 (1:250 dilution, ab255297, Abcam), anti‐MSMO1 (1:100 dilution, ab203587, Abcam), anti‐RPS14 (1:50 dilution, Abcam), anti‐FRZB (1:100 dilution, ab205284, Abcam), and anti‐MMP2 (1:200 dilution, ab86607, Abcam) antibodies overnight at 4 °C. The sections were then washed with PBS and incubated with a biotinylated secondary antibody for 15 min from a Histostain Plus kit (Invitrogen, CA, USA). The sections were then washed and incubated with 3, 3′‐diaminobenzidine for 2 min. Finally, using light microscopy to observe the section.

### Cell Sorting

Tissue samples were harvested from patients with IVDD and mechanically dissociated to generate single‐cell suspensions as described above. Cells were blocked with FcR Blocking Reagent, human (Miltenyi, 130‐059‐901) on ice for at least 10 min. Cells were then centrifuged at 300*g* for 5 min at 4 °C and washed once with BSA running buffer (0.5% BSA). Cells were incubated for 30 min at 4 °C with pre‐conjugated fluorescent labeled antibodies with the following combinations: CD45 (BioLegend, 368532 (PE/Cyanine7)), CD11b (BioLegend, 301330 (FITC)), OLR1 (BioLegend, 358604 (PE)), and CD24 (BioLegend, 311118 (APC)). Cells sorted by Beckman Moflo XDP and desired populations were isolated for different experiments. For CD90+ NPCs isolation, the primary cells were used within one passage. Cells were incubated with CD90 microbeads (Miltenyi Biotec, 130‐096‐253; 1 µL per 107 cells) to enrich CD90+ NPCs.

### Multilineage Differentiation Assays In Vitro

For osteogenic differentiation, the CD90+ NP cells were cultured in a humidified incubator (37 °C, 5% CO_2_) with renewal of the culture medium every 3 d. The cells were incubated in a differentiation medium for 2–4 weeks once the cells had reached 100% confluence, during which time the medium was changed every 2–3 d. The differentiation medium was as follows: DMEM‐LG supplemented with 10% FBS, 1 × 10^−6^ m dexamethasone (Sigma), 50 µg mL^−1^ ascorbic acid (Sigma), 10 × 10^−3^ m sodium *β*‐glycerophosphate (Sigma), and 1% penicillin/streptomycin (Sigma). The cells were fixed with ice‐cold 70% ethanol and stained with Alizarin Red S (Amresco, Solon, OH), as well as the von Kossa stain, to detect mineralization (calcium deposits). The alkaline phosphatase (ALP) activity was also tested. For adipogenic differentiation, the CD90+ cells were first grown to 100% confluence and then incubated for 3 d in an induction medium consisting of DMEM‐LG supplemented with 10% FBS, 100 × 10^−6^ m indomethacin (Sigma), 0.1 × 10^−6^ m dexamethasone, 0.5 × 10^−3^ m IBMX, 10 µg mL^−1^ human insulin, and 1% penicillin/streptomycin. The cells were incubated in the induction and maintenance media for >2 weeks and then fixed with 4% paraformaldehyde for 30 min at room temperature and stained with Oil Red O, as well as Sudan Black B, to detect fat deposition. For chondrogenic differentiation, cells were maintained as pellet cultures (2.5–5 × 105 cells/pellet) in DMEM high‐glucose medium supplemented with insulin transferin selenium (with albumin), sodium pyruvate (100 µg mL^−1^), l‐proline (40 µg mL^−1^), ascorbate 2‐phosphate (50 µg mL^−1^), and TGF‐*β*3 (10 ng mL^−1^).

### SDS‐PAGE Immunoblotting

SDS‐PAGE Immunoblotting was performed to analyze protein levels. The cells were lysed using RIPA lysis buffer, and protein concentrations were determined using the BCA assay. Nuclear and cytoplasmic proteins were lysed using the Nuclear/Cytosolic Fractionation assay kit from BioVision (Mountain View, CA) following the manufacturer's protocol. The membrane was blocked with 5% non‐fat milk and then incubated with the following primary antibodies at 4 °C overnight: anti‐BAX (1:1000), anti‐Bcl‐2 (1:1000), anti‐NLRP3 (1:1000), anti‐aggrecan (1:1000), and anti‐MMP‐13 (1:4000). After this incubation, the membranes were incubated for 2 h on a shaker at 37 °C with horseradish peroxidase (HRP)‐conjugated secondary antibodies (Boster, Wuhan, China) used at a dilution of 1: 2000 and then washed. Finally, the protein bands were visualized and detected using the enhanced chemiluminescence (ECL) system, and immunoreactive bands were quantified using the ImageQuant LAS 400 software (GE Healthcare Life Sciences) and calculated by normalization to the reference bands of GAPDH or lamin B.

### Histopathology and Immunofluorescence

For histopathological analysis, tissues were fixed in 10% formalin for 96 h, decalcified in 10% ethylenediaminetetraacetic acid (EDTA) for at least one month, embedded in paraffin, and 4–5 µm sections were stained with Safranin O/Fast Green Stain. For immunofluorescence, the caudal disc sections were blocked in 5% normal serum (Thermo Fisher Scientific, 10000 C) in PBS‐T (0.4% Triton X‐100 in PBS) and incubated with the primary antibody. The primary antibodies used were CD11b (1:500, 120772, Absin), OLR1(1:500, Absin, 123947), CD24 (1:200, Proteintech, 10600‐1‐AP). After washed with PBS/1% BSA, the sections were incubated with Alexa‐conjugated antibodies and washed with PBS/1% BSA followed by PBS. The DAPI was used for counterstaining.

### T‐Cell Suppression Assay

T‐lymphocytes were isolated from healthy donor's PBMCs via Pan T Cell Isolation Kit (Miltenyi Biotec, 130‐096‐535) following the manufacturer's instructions. Isolated T‐cells were then cultured in 96‐well round‐bottom plates in complete culture medium containing soluble, or plate‐bound, anti‐CD3 (1 µg mL^−1^) and soluble anti‐CD28 (5 µg mL^−1^). Sorted CD45+CD11b+OLR1+CD24+ and CD45+CD11b+OLR1+CD24− cells were added to T‐cells in 1:1 ratio. After 4 d of culture, T‐cell proliferation was assessed by MTT assay. T‐cells without sorted cells co‐cultured were used as positive control. Supernatants of the co‐culture were collected, the level of interleukin 2 (IL2) were measured by ELISA (Abcam) according to the manufacturer's protocol.

### ROS Production Assay

After isolating NP‐derived MDSCs by fluorescence‐activated cell sorting, the cellular ROS level was determined with the ROS assay kit. The cell suspension was used at a concentration of 1 × 10^6^ cells mL^−1^ and was diluted with DCFH‐DA solution (10 × 10^−6^ m) and incubated at 37 °C for 20 min with mixing by inversion every 5 min. After washing the cells with serum‐free medium, the sample was analyzed by flow cytometry (FAC Scan, Becton Dickenson, USA). Rosup was used as positive control.

## Conflict of Interest

The authors declare no conflict of interest.

## Author Contributions

J.T. and W.L. and S.D.Y. contributed equally to this work. J.T. conceptualized the project and planned, executed, and prepared the work for publication. J.Z. provided supports, supervised and initiated this study. J.T. performed most experiments, analyzed the scRNAseq data, and produced all figures and tables. W.T.L. and S.D.Y. performed part of the experiments and helped in data analysis. J.T. and W.T.L. wrote the manuscript with inputs from all authors. P.Y.Y. provided comments and feedback to the scRNA‐seq and manuscript. Q.Y. and C.H.W. collected samples and clinical data. K.T.L. and X.P.B. edited the manuscript. W.Y.D., J.C.W., A.D.D., C.Y., and H.L.Y. revised the manuscript, provided comments, and coordinated the collaboration.

## Supporting information

Supporting Information

## Data Availability

Our detailed single‐cell RNA‐seq data deposited at the NCBI's Gene expression omnibus (GEO) data repository with the accession ID GSE165722. Other data needed to evaluate the conclusions in the paper are present in the paper and/or the Supplementary Materials. Additional data related to this paper may be requested from the authors.

## References

[1] F. Balagué , A. F. Mannion , F. Pellisé , C. Cedraschi , Lancet 2012, 379, 482. 21982256 10.1016/S0140-6736(11)60610-7

[2] a) N. E. Foster , J. R. Anema , D. Cherkin , R. Chou , S. P. Cohen , D. P. Gross , P. H. Ferreira , J. M. Fritz , B. W. Koes , W. Peul , J. A. Turner , C. G. Maher , R. Buchbinder , J. Hartvigsen , D. Cherkin , N. E. Foster , C. G. Maher , M. Underwood , M. van Tulder , J. R. Anema , R. Chou , S. P. Cohen , L. Menezes Costa , P. Croft , M. Ferreira , P. H. Ferreira , J. M. Fritz , S. Genevay , D. P. Gross , M. J. Hancock , D. Hoy , J. Karppinen , B. W. Koes , A. Kongsted , Q. Louw , B. Öberg , W. C. Peul , G. Pransky , M. Schoene , J. Sieper , R. J. Smeets , J. A. Turner , A. Woolf , Lancet 2018, 391, 2368; 29573872 10.1016/S0140-6736(18)30489-6

[3] X. Cheng , L. Zhang , K. Zhang , G. Zhang , Y. Hu , X. Sun , C. Zhao , H. Li , Y. M. Li , J. Zhao , Ann. Rheum. Dis. 2018, 77, 770. 29343508 10.1136/annrheumdis-2017-212056 PMC5909753

[4] P. P. Vergroesen , I. Kingma , K. S. Emanuel , R. J. Hoogendoorn , T. J. Welting , B. J. van Royen , J. H. van Dieën , T. H. Smit , Osteoarthritis Cartilage 2015, 23, 1057. 25827971 10.1016/j.joca.2015.03.028

[5] a) P. Heindel , A. Tuchman , P. C. Hsieh , M. H. Pham , A. D'Oro , N. N. Patel , A. M. Jakoi , R. Hah , J. C. Liu , Z. Buser , J. C. Wang , Spine 2017, 42, E496; 27548580 10.1097/BRS.0000000000001855

[6] H. Morris , C. F. Gonçalves , M. Dudek , J. Hoyland , Q. J. Meng , Ann. Rheum. Dis. 2021, 10.1136/annrheumdis-2020-219515. 33397731

[7] R. M. Ransohoff , M. A. Brown , J. Clin. Invest. 2012, 122, 1164. 22466658 10.1172/JCI58644 PMC3314450

[8] a) H. Sun , X. Wen , H. Li , P. Wu , M. Gu , X. Zhao , Z. Zhang , S. Hu , G. Mao , R. Ma , W. Liao , Z. Zhang , Ann. Rheum. Dis. 2020, 79, 408 31871141 10.1136/annrheumdis-2019-215926 PMC7034356

[9] a) J. Buckland , Nat. Rev. Rheumatol. 2012, 8, 368; 22641139 10.1038/nrrheum.2012.82

[10] L. Liu , X. Liu , H. Cui , R. Liu , G. Zhao , J. Wen , BMC Genomics 2019, 20, 863. 31729950 10.1186/s12864-019-6221-0 PMC6858653

[11] X. Y. Lu , X. J. Shi , A. Hu , J. Q. Wang , Y. Ding , W. Jiang , M. Sun , X. Zhao , J. Luo , W. Qi , B. L. Song , Nature 2020, 588, 479. 33177714 10.1038/s41586-020-2928-y

[12] S. C. Sun , Nat. Rev. Immunol. 2017, 17, 545. 28580957 10.1038/nri.2017.52 PMC5753586

[13] A. J. Zollinger , M. L. Smith , Matrix Biol. 2017, 60, 27. 27496349 10.1016/j.matbio.2016.07.011

[14] Y. Mao , J. E. Schwarzbauer , Matrix Biol. 2005, 24, 389. 16061370 10.1016/j.matbio.2005.06.008

[15] S. Zhongyi , Z. Sai , L. Chao , T. Jiwei , Spine 2015, 40. 10.1097/BRS.0000000000000733 25494317

[16] S. Chen , S. Liu , K. Ma , L. Zhao , H. Lin , Z. Shao , Osteoarthritis Cartilage 2019, 27, 1109. 31132405 10.1016/j.joca.2019.05.005

[17] L. Li , J. Zou , Y. Dai , W. Fan , G. Niu , Z. Yang , X. Chen , Nat. Biomed. Eng. 2020, 4, 1102. 32807941 10.1038/s41551-020-0599-5

[18] a) G. Yaari , C. R. Bolen , J. Thakar , S. H. Kleinstein , Nucleic Acids Res. 2013, 41, e170; 23921631 10.1093/nar/gkt660 PMC3794608

[19] a) Y. C. Huang , J. P. Urban , K. D. Luk , Nat. Rev. Rheumatol. 2014, 10, 561; 24914695 10.1038/nrrheum.2014.91

[20] G. S. Gulati , S. S. Sikandar , D. J. Wesche , A. Manjunath , A. Bharadwaj , M. J. Berger , F. Ilagan , A. H. Kuo , R. W. Hsieh , S. Cai , M. Zabala , F. A. Scheeren , N. A. Lobo , D. Qian , F. B. Yu , F. M. Dirbas , M. F. Clarke , A. M. Newman , Science 2020, 367, 405. 31974247 10.1126/science.aax0249 PMC7694873

[21] V. Bergen , M. Lange , S. Peidli , F. A. Wolf , F. J. Theis , Nat. Biotechnol. 2020, 38, 1408. 32747759 10.1038/s41587-020-0591-3

[22] K. R. Jacobson , S. Lipp , A. Acuna , Y. Leng , Y. Bu , S. Calve , J. Proteome Res. 2020, 19, 3955. 32830507 10.1021/acs.jproteome.0c00248 PMC8325396

[23] J. M. Moreno‐Navarrete , M. G. Novelle , V. Catalán , F. Ortega , M. Moreno , J. Gomez‐Ambrosi , G. Xifra , M. Serrano , E. Guerra , W. Ricart , G. Frühbeck , C. Diéguez , J. M. Fernández‐Real , Diabetes Care 2014, 37, 1092. 24496804 10.2337/dc13-1602

[24] J. Roth , M. Goebeler , C. Sorg , Lancet 2001, 357, 1041. 10.1016/S0140-6736(05)71610-X 11293617

[25] A. Bergmann , H. Steller , Sci. Signaling 2010, 3, re8. 10.1126/scisignal.3145re8 PMC2991142 20978240

[26] a) D. R. Green , Cell 2019, 177, 1094; 31100266 10.1016/j.cell.2019.04.024 PMC6534278

[27] A. Butler , P. Hoffman , P. Smibert , E. Papalexi , R. Satija , Nat. Biotechnol. 2018, 36, 411. 29608179 10.1038/nbt.4096 PMC6700744

[28] J. Yu , H. C. Mao , M. Wei , T. Hughes , J. Zhang , I. K. Park , S. Liu , S. McClory , G. Marcucci , R. Trotta , M. A. Caligiuri , Blood 2010, 115, 274. 19897577 10.1182/blood-2009-04-215491 PMC2808153

[29] S. Thornton , R. Tan , A. Sproles , T. Do , J. Schick , A. A. Grom , M. DeLay , G. S. Schulert , J. Immunol. 2019, 202, 1635. 30683706 10.4049/jimmunol.1800765 PMC6382590

[30] a) Z. Liu , Y. Gu , S. Chakarov , C. Bleriot , I. Kwok , X. Chen , A. Shin , W. Huang , R. J. Dress , C. A. Dutertre , A. Schlitzer , J. Chen , L. G. Ng , H. Wang , Z. Liu , B. Su , F. Ginhoux , Cell 2019, 178, 1509; 31491389 10.1016/j.cell.2019.08.009

[31] N. Meknache , F. Jönsson , J. Laurent , M. T. Guinnepain , M. Daëron , J. Immunol. 2009, 182, 2542. 19201911 10.4049/jimmunol.0801665

[32] a) C. Perez , C. Botta , A. Zabaleta , N. Puig , M. T. Cedena , I. Goicoechea , D. Alameda , E. San José‐Eneriz , J. Merino , P. Rodríguez‐Otero , C. Maia , D. Alignani , P. Maiso , I. Manrique , D. Lara‐Astiaso , A. Vilas‐Zornoza , S. Sarvide , C. Riillo , M. Rossi , L. Rosiñol , A. Oriol , M. J. Blanchard , R. Rios , A. Sureda , J. Martin , R. Martinez , J. Bargay , J. de la Rubia , M. T. Hernandez , J. Martinez‐Lopez , A. Orfao , X. Agirre , F. Prosper , M. V. Mateos , J. J. Lahuerta , J. Blade , J. F. San‐Miguel , B. Paiva , Blood 2020, 136, 199; 32325491 10.1182/blood.2019004537

[33] F. Veglia , E. Sanseviero , D. I. Gabrilovich , Nat. Rev. Immunol. 2021, 21, 485. 33526920 10.1038/s41577-020-00490-y PMC7849958

[34] a) K. Ohl , K. Tenbrock , Front. Immunol. 2018, 9, 2499; 30425715 10.3389/fimmu.2018.02499 PMC6218613

[35] L. W. Collison , D. A. A. Vignali , Methods Mol. Biol. 2011, 707, 21. 21287326 10.1007/978-1-61737-979-6_2 PMC3043080

[36] K. L. Phillips , N. Jordan‐Mahy , M. J. Nicklin , C. L. Le Maitre , Ann. Rheum. Dis. 2013, 72, 1860. 23396662 10.1136/annrheumdis-2012-202266

[37] M. Efremova , M. Vento‐Tormo , S. A. Teichmann , R. Vento‐Tormo , Nat. Protoc. 2020, 15, 1484. 32103204 10.1038/s41596-020-0292-x

[38] Y. Koike , M. Uzuki , S. Kokubun , T. Sawai , Spine 2003, 28. 10.1097/01.BRS.0000083324.65405.AE 12973136

[39] D. W. McMillan , G. Garbutt , M. A. Adams , Ann. Rheum. Dis. 1996, 55, 880. 9014581 10.1136/ard.55.12.880 PMC1010338

[40] M. Calió , B. Gantenbein , M. Egli , L. Poveda , F. Ille , Int. J. Mol. Sci. 2021, 22, 4917. 34066404 10.3390/ijms22094917 PMC8124861

[41] P. M. van der Kraan , W. B. van den Berg , Osteoarthritis Cartilage 2012, 20, 223. 22178514 10.1016/j.joca.2011.12.003

[42] a) J. P. Rutges , R. A. Duit , J. A. Kummer , F. C. Oner , M. H. van Rijen , A. J. Verbout , R. M. Castelein , W. J. Dhert , L. B. Creemers , Osteoarthritis Cartilage 2010, 18, 1487; 20723612 10.1016/j.joca.2010.08.006

[43] N. Nakayama , C. Y. Han , L. Cam , J. I. Lee , J. Pretorius , S. Fisher , R. Rosenfeld , S. Scully , R. Nishinakamura , D. Duryea , G. Van , B. Bolon , T. Yokota , K. Zhang , Development 2004, 131, 229. 14660436 10.1242/dev.00901

[44] a) D. Sakai , J. Schol , F. C. Bach , A. Tekari , N. Sagawa , Y. Nakamura , S. C. W. Chan , T. Nakai , L. B. Creemers , D. A. Frauchiger , R. D. May , S. Grad , M. Watanabe , M. A. Tryfonidou , B. Gantenbein , JOR Spine 2018, 1, e1018; 31463445 10.1002/jsp2.1018 PMC6686801

[45] D. Sakai , Y. Nakamura , T. Nakai , T. Mishima , S. Kato , S. Grad , M. Alini , M. V. Risbud , D. Chan , K. S. Cheah , K. Yamamura , K. Masuda , H. Okano , K. Ando , J. Mochida , Nat. Commun. 2012, 3, 1264. 23232394 10.1038/ncomms2226 PMC3535337

[46] K. Siemionow , H. An , K. Masuda , G. Andersson , G. Cs‐Szabo , Spine 2011, 36, 1333. 21217432 10.1097/BRS.0b013e3181f2a177 PMC3117081

[47] J. F. Blanco , I. F. Graciani , F. M. Sanchez‐Guijo , S. Muntión , P. Hernandez‐Campo , C. Santamaria , S. Carrancio , M. V. Barbado , G. Cruz , S. Gutierrez‐Cosío , C. Herrero , J. F. San Miguel , J. G. Briñon , M. C. del Cañizo , Spine 2010, 35, 2259. 20622750 10.1097/BRS.0b013e3181cb8828

[48] a) H. D. Ryoo , T. Gorenc , H. Steller , Dev. Cell 2004, 7, 491; 15469838 10.1016/j.devcel.2004.08.019

[49] J. Folkman , Curr. Mol. Med. 2003, 3, 643. 14601638 10.2174/1566524033479465

[50] H. Henriksson , M. Thornemo , C. Karlsson , O. Hägg , K. Junevik , A. Lindahl , H. Brisby , Spine 2009, 34, 2278. 19755937 10.1097/BRS.0b013e3181a95ad2

[51] Z. Sun , B. Liu , Z.‐J. Luo , Int. J. Med. Sci. 2020, 17, 685. 32210719 10.7150/ijms.42238 PMC7085207

[52] A. L. A. Binch , A. A. Cole , L. M. Breakwell , A. L. R. Michael , N. Chiverton , L. B. Creemers , A. K. Cross , C. L. Le Maitre , Arthritis Res. Ther. 2015, 17, 370. 26695177 10.1186/s13075-015-0889-6 PMC4704545

[53] A. J. Freemont , A. Watkins , C. Le Maitre , P. Baird , M. Jeziorska , M. T. N. Knight , E. R. S. Ross , J. P. O'Brien , J. A. Hoyland , J. Pathol. 2002, 197, 286. 12115873 10.1002/path.1108

[54] R. Ali , C. L. Le Maitre , S. M. Richardson , J. A. Hoyland , A. J. Freemont , Biotech. Histochem. 2008, 83, 239. 19016368 10.1080/10520290802539186

[55] G. David , A. V. Ciurea , S. M. Iencean , A. Mohan , J. Med. Life 2010, 3, 154. 20968201 PMC3019053

[56] A. J. Freemont , A. Watkins , C. Le Maitre , M. Jeziorska , J. A. Hoyland , J. Pathol. 2002, 196, 374. 11920731 10.1002/path.1050

[57] a) M. Evrard , I. W. H. Kwok , S. Z. Chong , K. W. W. Teng , E. Becht , J. Chen , J. L. Sieow , H. L. Penny , G. C. Ching , S. Devi , J. M. Adrover , J. L. Y. Li , K. H. Liong , L. Tan , Z. Poon , S. Foo , J. W. Chua , I. H. Su , K. Balabanian , F. Bachelerie , S. K. Biswas , A. Larbi , W. Y. K. Hwang , V. Madan , H. P. Koeffler , S. C. Wong , E. W. Newell , A. Hidalgo , F. Ginhoux , L. G. Ng , Immunity 2018, 48, 364; 29466759 10.1016/j.immuni.2018.02.002

[58] a) F. Veglia , M. Perego , D. Gabrilovich , Nat. Immunol. 2018, 19, 108; 29348500 10.1038/s41590-017-0022-x PMC5854158

[59] D. I. Gabrilovich , S. Nagaraj , Nat. Rev. Immunol. 2009, 9, 162. 19197294 10.1038/nri2506 PMC2828349

[60] L. A. Haile , R. von Wasielewski , J. Gamrekelashvili , C. Krüger , O. Bachmann , A. M. Westendorf , J. Buer , R. Liblau , M. P. Manns , F. Korangy , T. F. Greten , Gastroenterology 2008, 135, 871. 18674538 10.1053/j.gastro.2008.06.032

[61] N. Fujita , T. Miyamoto , J. Imai , N. Hosogane , T. Suzuki , M. Yagi , K. Morita , K. Ninomiya , K. Miyamoto , H. Takaishi , M. Matsumoto , H. Morioka , H. Yabe , K. Chiba , S. Watanabe , Y. Toyama , T. Suda , Biochem. Biophys. Res. Commun. 2005, 338, 1890. 16288985 10.1016/j.bbrc.2005.10.166

[62] Z. Liu , Z. Zheng , J. Qi , J. Wang , Q. Zhou , F. Hu , J. Liang , C. Li , W. Zhang , X. Zhang , J. Biol. Eng. 2018, 12, 35. 30598696 10.1186/s13036-018-0129-0 PMC6303933

[63] S. M. Richardson , F. E. Ludwinski , K. K. Gnanalingham , R. A. Atkinson , A. J. Freemont , J. A. Hoyland , Sci. Rep. 2017, 7, 1501. 28473691 10.1038/s41598-017-01567-w PMC5431421

[64] N. Fujita , T. Miyamoto , J.‐I. Imai , N. Hosogane , T. Suzuki , M. Yagi , K. Morita , K. Ninomiya , K. Miyamoto , H. Takaishi , M. Matsumoto , H. Morioka , H. Yabe , K. Chiba , S. Watanabe , Y. Toyama , T. Suda , Biochem. Biophys. Res. Commun. 2005, 338, 1890. 16288985 10.1016/j.bbrc.2005.10.166

[65] a) M. Hubbe , P. Altevogt , Eur. J. Immunol. 1994, 24, 731; 8125140 10.1002/eji.1830240336

[66] S. Chen , Y. Zhou , Y. Chen , J. Gu , Bioinformatics 2018, 34, i884. 30423086 10.1093/bioinformatics/bty560 PMC6129281

