# Supplementary material for: Single‐Cell Transcriptome Profiling Reveals Multicellular Ecosystem of Nucleus Pulposus during Degeneration Progression
Source: Adv Sci (Weinh). 2021 Nov 26;9(3):2103631. doi: 10.1002/advs.202103631 (PMC8787427; doi:10.1002/advs.202103631)

ADVANCED SCIENCE
Open Access

Supporting Information

for *Adv. Sci.*, DOI: 10.1002/advs.202103631

Single-cell transcriptome profiling reveals multicellular ecosystem of nucleus pulposus during degeneration progression

*Ji Tu†, Wentian Li†, Sidong Yang†, Pengyi Yang, Qi Yan, Shenyu Wang, Kaitao Lai, Xupeng Bai, Cenhao Wu, Wenyuan Ding, Justin Cooper-White, Ashish Diwan, Cao Yang, Huilin Yang, Jun Zou**

Supplementary Figures

Figure S1. (A)The stage of generation was based on IVD structure and MRI signal intensity. (B-C) Cell quality for single cell sequencing. Hematoxylin and eosin (H&E) staining showed structure of the human disc(B), the NP (C) and the AF(D). Scare bar, 5mm in A; 200um in B and C. (D-H) PCA and tSNE plots of all 39,732 single-cell transcriptomes colored by individual participants.

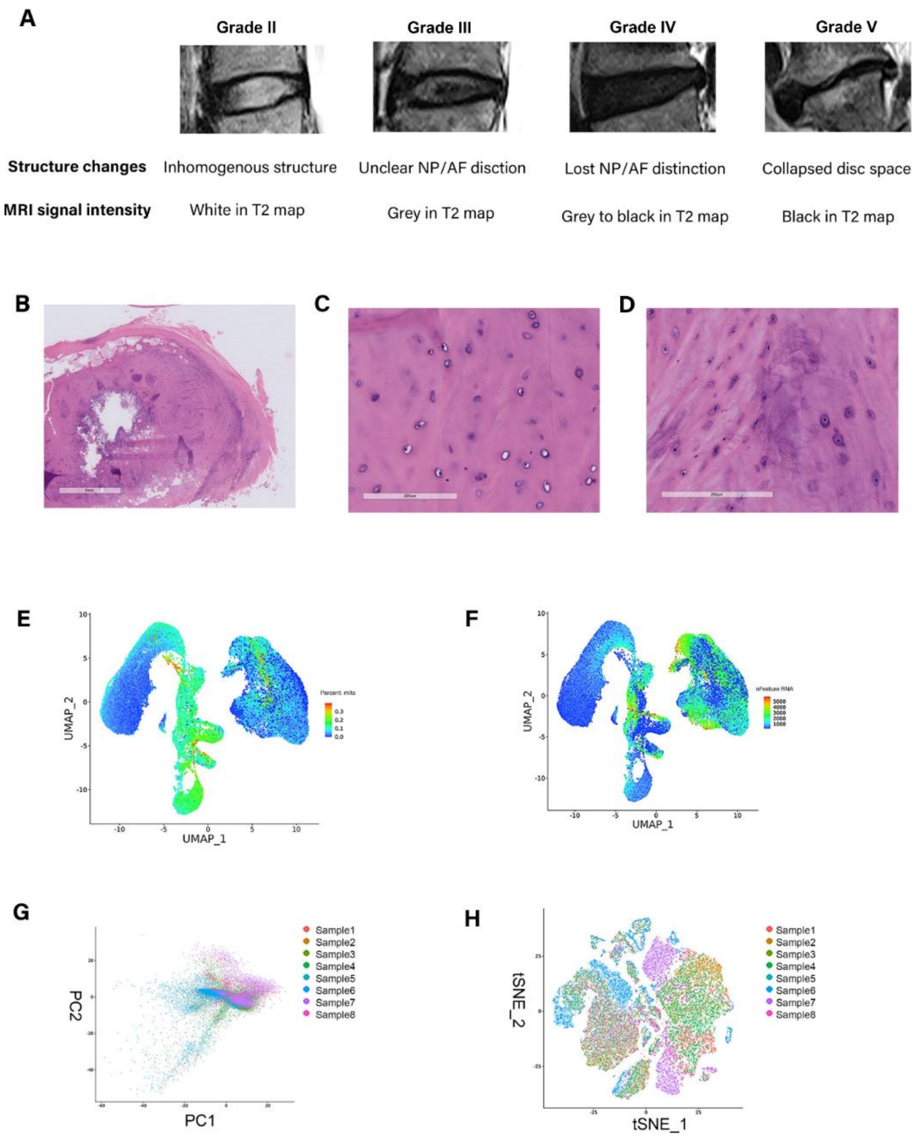

Figure S2. (A) Heatmap showed the area under the curve (AUC) scores of TF estimated per cell. Top three differentially activated transcription factor in each NPCs subpopulations were showed. (B) tSNE map showed the expression of the TFs and their targets AUC scores.

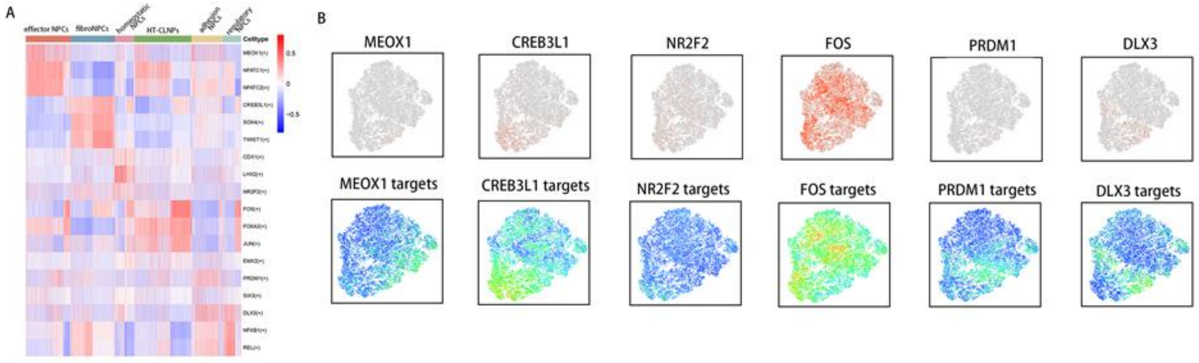

Figure S3. (A) Monocle method reconstruction of pseudospace trajectory for defined NP cell clusters. (B) Identify putative driver genes for fibroNPCs by scVelo.

A

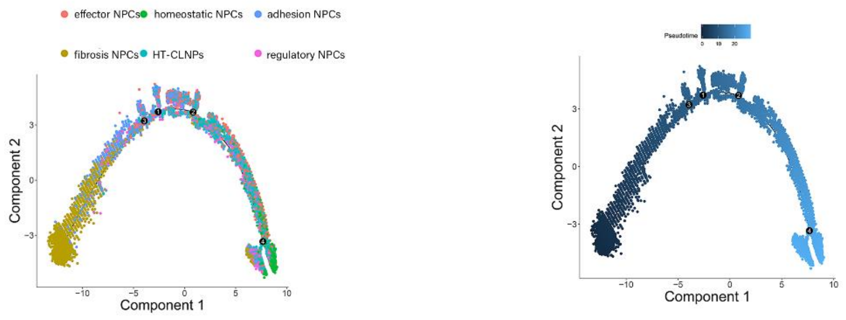

B

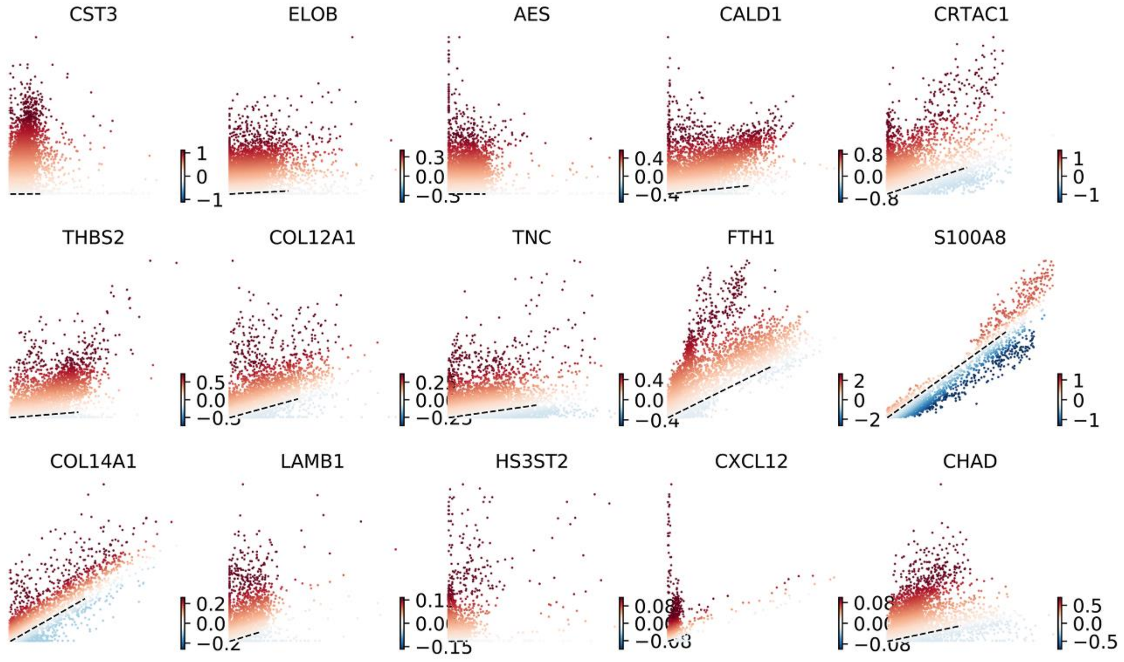

Figure S4. (A) Gating strategies for flow sorting of NP-derived MDSCs. (B) Levels of IL2 were determined in supernatants of T cells activated by coated anti-CD3/CD28 antibodies with and without NP derived G-MDSCs after 4 days. Relative release was calculated to stimulated T cell without NP derived G-MDSCs. (n = 8–10 for each group) Mean ± SD is reported. Student's t test was applied. Results were considered significant at *, $P \leq 0.05$; **, $P \leq 0.005$; and ***, $P \leq 0.0005$.

A

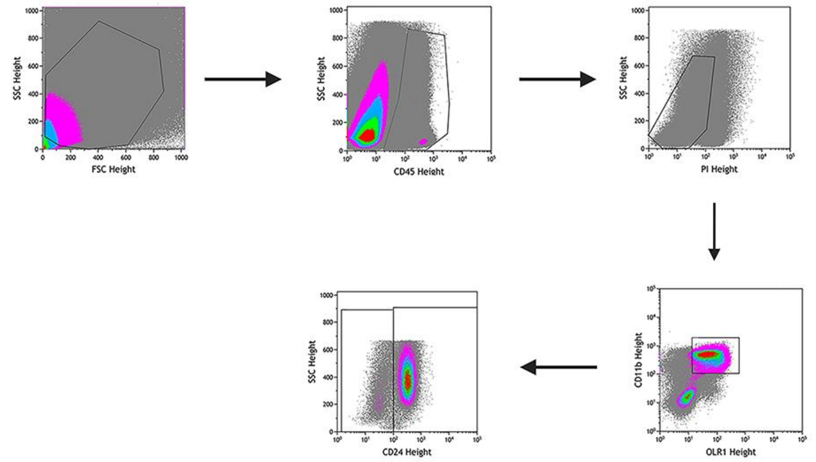

B

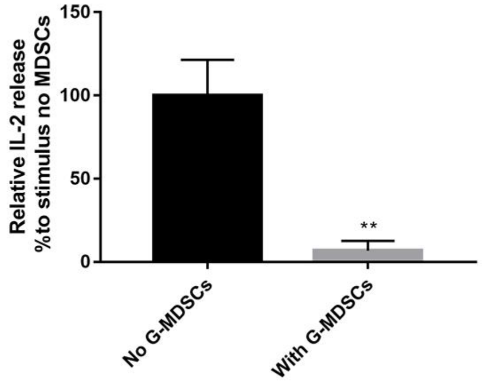

Figure S5 (A) Expression of CD44, CD29, CD34, and HLA-DR in CD90+NPCs. (B)Expression of TEK (Tie2) and Gd2 in human NPCs in UMAP. (B) Schematic graph of the potential hypothetical cellular migration pathways. NP: Nucleus pulposus, AF: Anulus fibrosus.

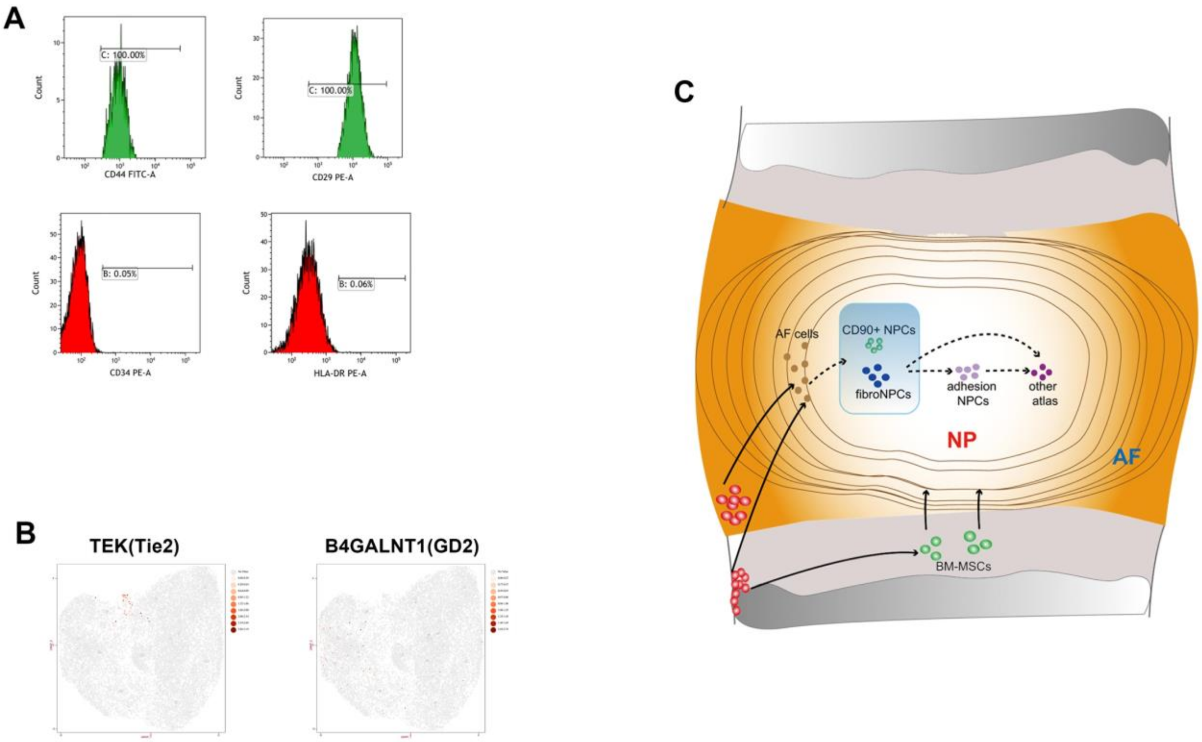

Supplement: Supplementary file 1 — Supporting Information [file ADVS-9-2103631-s001.pdf]
